# Effects of high-intensity interval training on physical fitness in trained adolescent athletes: a systematic review and meta-analysis

**DOI:** 10.3389/fphys.2026.1839190

**Published:** 2026-05-18

**Authors:** Fengming Zhang, Yang Liu, Jiale Liu, Cheng Li, Zengjing Liu, Oleksandr Yeremenko

**Affiliations:** 1 National University of Physical Education and Sport of Ukraine, Kyiv, Ukraine; 2 Taishan College of Science and Technology, Taian, China

**Keywords:** Adolescent athletes, athletic performance, high-intensity interval training, physical fitness, repeated sprint training, sprint interval training

## Abstract

**Background:**

This systematic review and meta-analysis evaluated the effects of HIIT-based interventions on cardiorespiratory fitness, jump, sprint, change-of-direction, and repeated sprint ability in trained adolescent athletes, and explored potential moderators of these effects.

**Methods:**

PubMed, Scopus, Embase, Web of Science, and the Cochrane Library were systematically searched. Between-group effect sizes were calculated from pre-to-post changes and pooled using random-effects models. Risk of bias and certainty of evidence were assessed using the Cochrane RoB 2 tool and the GRADE approach. Heterogeneity was assessed using I², and prespecified subgroup, sensitivity, and meta-regression analyses were performed when appropriate.

**Results:**

Thirty-five studies involving 988 trained adolescent athletes were included. Compared with non-HIIT control conditions, HIIT-based interventions significantly improved VO_2_max (SMD = 0.65, 95% CI 0.46 to 0.83, I² = 0%), field-based intermittent endurance test performance, including Yo-Yo IR1, Yo-Yo IR2, and the 20m multistage fitness test (SMD = 0.65, 95% CI 0.07 to 1.23, I² = 79.4%), final velocity reached in the 30–15 Intermittent Fitness Test (VIFT) (SMD = 1.13, 95% CI 0.63 to 1.63, I² = 42.5%), countermovement jump (CMJ) (SMD = 0.44, 95% CI 0.13 to 0.74, I² = 62.7%), ≤10m sprint performance (SMD = -0.79, 95% CI -1.26 to -0.32, I² = 50.1%), ≥20m sprint performance (SMD = -0.28, 95% CI -0.47 to -0.08, I² = 0%), and change-of-direction (COD) (SMD = -0.54, 95% CI -0.72 to -0.37, I² = 0%). For repeated sprint ability (RSA), all included interventions were repeated sprint training (RST), and the pooled effect was significant (SMD = -0.70, 95% CI -1.02 to -0.39, I² = 12.7%). Significant between-subgroup differences were found only for age in field-based intermittent endurance test performance (p = 0.033) and competitive level in ≥20m sprint performance (p = 0.034), although these subgroup findings should be interpreted as exploratory.

**Conclusion:**

HIIT-based interventions may improve multiple physical fitness outcomes in trained adolescent athletes, with larger effects observed for VO_2_max, field-based intermittent endurance test performance, VIFT, and ≤10m sprint. Improvements in CMJ, ≥20m sprint, and COD were smaller. For RSA, current evidence mainly supports a beneficial effect of RST.

**Systematic review registration:**

https://www.crd.york.ac.uk/PROSPERO/, identifier CRD420261348570.

## 1 Introduction

Adolescence is a critical period for the development of physical fitness and athletic performance, during which youth athletes undergo substantial growth and maturation-related changes that can influence training adaptation and athletic performance (Bergeron et al., 2015; Lloyd et al., 2015, Lloyd et al., 2014). High levels of cardiorespiratory fitness, intermittent endurance, jumping, sprinting, change-of-direction (COD), and repeated sprint ability (RSA) are not only important for training quality but also closely associated with competitive level and match performance (Girard and Millet, 2009; Madsen et al., 2018; Ortega-Becerra et al., 2020; Rebelo et al., 2014). Because youth athletes are still undergoing growth and development while also receiving long-term systematic training, their responses to training may differ from those of untrained adolescents and adult athletes (Behringer et al., 2010; Dotan et al., 2012). In addition, adolescent athletes often need to balance sport-specific and physical training within limited training time. Therefore, it is particularly important to select training methods that are both efficient and practically applicable (Bergeron et al., 2024).

High-intensity interval training (HIIT) is usually performed at near-maximal, maximal, or supramaximal exercise intensity and consists of short periods of high-intensity exercise interspersed with brief passive or active recovery periods (Buchheit and Laursen, 2013; MacInnis and Gibala, 2017; Tschakert and Hofmann, 2013). Previous studies have shown that HIIT-based interventions can significantly improve multiple components of physical fitness, including cardiorespiratory fitness, intermittent endurance, jumping, sprinting, and COD (Engel et al., 2018; Stankovic et al., 2023; Yuan et al., 2024). Accordingly, HIIT-based interventions are widely used in modern sports training. However, HIIT-based interventions do not represent a single training format (Buchheit and Laursen, 2013). According to differences in exercise intensity, movement characteristics, and duration, they can be classified into short-interval training, long-interval training, sprint interval training (SIT), and repeated sprint training (RST) (Bucheit and Laursen, 2013; Coates et al., 2023; Girard et al., 2011). Moreover, it should be noted that different HIIT types may differ in their training stimulus characteristics and physical adaptations, and may therefore produce different effects on different physical fitness outcomes (Bishop et al., 2011; Buchheit and Laursen, 2013; Rosenblat et al., 2020).

Although previous reviews have generally reported that HIIT-based interventions can improve cardiorespiratory fitness and some physical fitness outcomes in adolescent or athletic populations, the current evidence remains insufficient for trained adolescent athletes specifically. Some reviews have combined untrained individuals, non-athlete adolescents, and adults within the same analysis, which limits the specificity of the conclusions for trained adolescent athletes (Costigan et al., 2015; Eddolls et al., 2017; Weston et al., 2014; Wiesinger et al., 2025). Moreover, previous reviews have focused mainly on a single sport or only some physical fitness outcomes, and have lacked systematic integration of multiple physical fitness outcomes in trained adolescent athletes (Clemente et al., 2024; Kunz et al., 2019; Manuel Clemente et al., 2021).

Therefore, this systematic review and meta-analysis aimed to evaluate the effects of HIIT-based interventions on cardiorespiratory fitness, jump performance, sprint performance, change-of-direction performance, and repeated sprint ability in trained adolescent athletes. Specifically, the outcomes of interest included VO_2_max, field-based intermittent endurance test performance, including Yo-Yo IR1, Yo-Yo IR2, and the 20m multistage fitness test, final velocity reached in the 30–15 Intermittent Fitness Test (VIFT), countermovement jump (CMJ), sprint performance, change-of-direction speed (COD), and repeated sprint ability (RSA). Prespecified subgroup analyses were also conducted to explore potential participant and intervention characteristics associated with the training effects. These analyses were intended to provide more targeted evidence for optimizing physical training programs in trained adolescent athletes.

## 2 Methods

### 2.1 Protocol and registration

This systematic review was conducted and presented in line with PRISMA 2020 (Page et al., 2021), and its protocol was registered in PROSPERO in advance (CRD420261348570).

### 2.2 Literature search strategy

A systematic search was conducted in five electronic databases, namely PubMed, Scopus, Embase, Web of Science, and the Cochrane Library, up to February 26, 2026. Only peer-reviewed studies published in English were included. The reference lists of relevant articles were also screened to make the review as comprehensive as possible. The full search strategy is presented in **Supplementary Table 1**.

### 2.3 Inclusion and exclusion criteria

The inclusion and exclusion criteria were established based on the PICOS framework, as shown in **Table 1**. Eligible HIIT-based interventions were defined as structured protocols involving repeated high-intensity work bouts and planned recovery intervals. Interventions were further categorized, where appropriate, as interval-type HIIT, SIT, or RST according to key protocol characteristics, including exercise intensity, interval duration, number of intervals, and overall intervention duration. These interventions were pooled within the overall HIIT-based framework because they all involved repeated high-intensity efforts interspersed with planned recovery periods. Adolescence was defined as 10–19 years (Organization, W.H, 2023). Studies were eligible when participants fell within this age range, or when data for adolescent participants could be extracted separately.

**Table 1:** Inclusion and exclusion criteria.

Category	Inclusion criteria	Exclusion criteria
Population (P)	Trained adolescent athletes classified as McKay Tier 2 or above, aged generally 10–19 years.	Untrained populations, general students or sedentary individuals, adult athletes, studies involving participants classified below Tier 2 or with undeterminable competitive level according to the McKay framework, and rehabilitation populations.
Intervention (I)	Interventions consisting of high-intensity interval training, including traditional HIIT, SIT, RST, and sport-specific HIIT programs, with a clearly defined intermittent high-intensity structure.	Non-HIIT interventions, high-intensity training without a clear interval structure, or training programs that could not be classified as HIIT.
Comparison (C)	Regular sport-specific training, coordination training, moderate- to low-intensity continuous training, resistance training, and other non-HIIT training programs.	Studies without a control group, or those in which the control group also performed HIIT, SIT, RST, or other high-intensity interval training.
Outcome (O)	Studies reporting at least one physical fitness outcome, such as VO_2_max, Yo-Yo IR1, Yo-Yo IR2, 30–15 IFT (VIFT), CMJ, SJ, sprint performance, COD, or RSA, with sufficient data for effect size calculation.	Studies that did not report relevant physical fitness outcomes or did not provide sufficient data for effect size calculation.
Study design (S)	Randomized controlled trials and non-randomized controlled trials.	Non-controlled studies, single-group pre-post studies, observational studies, systematic reviews, and meta-analyses.

In this study, trained adolescent athletes were defined as participants classified as Tier 2 or above according to the McKay Participant Classification Framework (MPCF) (McKay et al., 2021).

### 2.4 Literature screening and data extraction

All relevant records identified through the search were imported into EndNote 21 for duplicate removal. Thereafter, two authors (YL and JL) independently screened the titles, abstracts, and full texts. Any disagreements arising during the screening process were resolved through discussion and, when necessary, adjudicated by a third author (FZ).

Data extraction was independently completed by two authors (JZ and JL) using a predesigned standardized form. The extracted information included basic study characteristics, including the author and year of publication, sport, sex, age, sample size, competitive level, control intervention, study design, and outcome measures. In addition, the main characteristics of the intervention protocols were extracted, such as HIIT type, HIIT mode, intervention duration, training frequency, and load arrangement. Participants’ competitive level was classified according to the MPCF. For outcome data, the sample sizes, means, and standard deviations for each group before and after the intervention were extracted, along with other data that could be used to calculate effect sizes. If outcome data were missing or reported only in graphical form, the corresponding authors were contacted to obtain the required data. Any inconsistencies in the data extraction process were resolved through discussion and, when necessary, adjudicated by a third author (FZ).

### 2.5 Risk of bias and certainty of evidence assessment

Two authors (YL and CL) independently assessed the risk of bias of the included studies using the Cochrane RoB 2 tool. The domains assessed included the randomization process, deviations from intended interventions, missing outcome data, measurement of the outcome, and selection of the reported result (Sterne et al., 2019). Each domain and the overall risk of bias were judged as low risk, some concerns, or high risk. The two authors cross-checked the assessment results. Any disagreements were first resolved through discussion and, when necessary, adjudicated by a third author (FZ).

Two authors used the GRADE approach to assess the certainty of evidence for each outcome. According to risk of bias, inconsistency, indirectness, imprecision, and publication bias, the certainty of evidence was rated as high, moderate, low, or very low (Guyatt et al., 2011). Any disagreements were first resolved through discussion and, when necessary, adjudicated by a third author (FZ).

### 2.6 Statistical analysis

Meta-analyses were conducted using Stata 15.0. To account for possible baseline differences across studies, between-group effect sizes were calculated from pre-to-post changes. Change scores were defined as Mean_change_ = Mean_post_ − Mean_pre_ for each group. If the standard deviation of the change score was not directly reported, SD_change_ was estimated under the assumption of a correlation coefficient of r = 0.5 according to the formula SD_change_ = √(SD_pre_² + SD_post_² − 2r × SD_pre_ × SD_post_) (Higgins and Green, 2008). Because differences may exist across studies in participant characteristics, intervention features, and testing procedures, a random-effects model was used to pool effect sizes, expressed as standardized mean differences (SMDs) (Deeks et al., 2019). Hedges’ g was used to correct for small-sample bias, and the results were presented with 95% confidence intervals (95% CIs) (Higgins and Green, 2008). To avoid double-counting, when multiple similar indicators for the same outcome were reported in a single study, only one representative indicator was extracted. When one control group was compared with multiple experimental groups within the same study, the control group sample size was evenly distributed across comparisons.

Effect sizes were interpreted using the following thresholds: trivial (<0.2), small (0.2–0.6), moderate (>0.6–1.2), large (>1.2–2.0), very large (>2.0–4.0), and extremely large (>4.0) (Hopkins et al., 2009). Between-study heterogeneity was assessed using the I² statistic and categorized as low (<25%), moderate (25%–50%), high (50%–75%), or very high (>75%) (Higgins and Thompson, 2002).

When heterogeneity was high, prespecified subgroup analyses and sensitivity analyses were conducted to explore potential sources of heterogeneity. Subgroup analyses were primarily conducted by sex, age, intervention duration, training frequency, total number of training sessions, competitive level, HIIT type, and HIIT mode. Comparator-based subgroup analyses were not performed because comparator conditions were diverse across studies, and the number of studies within individual comparator categories was limited and uneven across outcomes. In addition, some subgroup analyses were conducted as exploratory to compare trends in effect size changes across conditions. Among these variables, continuous variables such as age, intervention duration, training frequency, and total number of training sessions were stratified using the median-split method. In subgroup analyses, HIIT type was classified as interval-type HIIT, SIT, and RST. Because the number of included studies in the short-interval, long-interval, and short-/long-interval HIIT subgroups were small, these subgroups were combined into an interval-type HIIT subgroup to improve the stability and interpretability of the subgroup analyses. Exploratory univariable meta-regression analyses were planned for outcomes with sufficient study numbers and substantial heterogeneity, with age, intervention duration, and training frequency each examined separately as continuous variables. Sensitivity analyses were performed using a leave-one-out approach to examine the robustness of the pooled results. Publication bias was assessed using Egger’s test when at least 10 studies were available for a given outcome. All tests were two-sided, and statistical significance was set at p < 0.05.

## 3 Results

### 3.1 Literature search and screening

**Figure 1:**
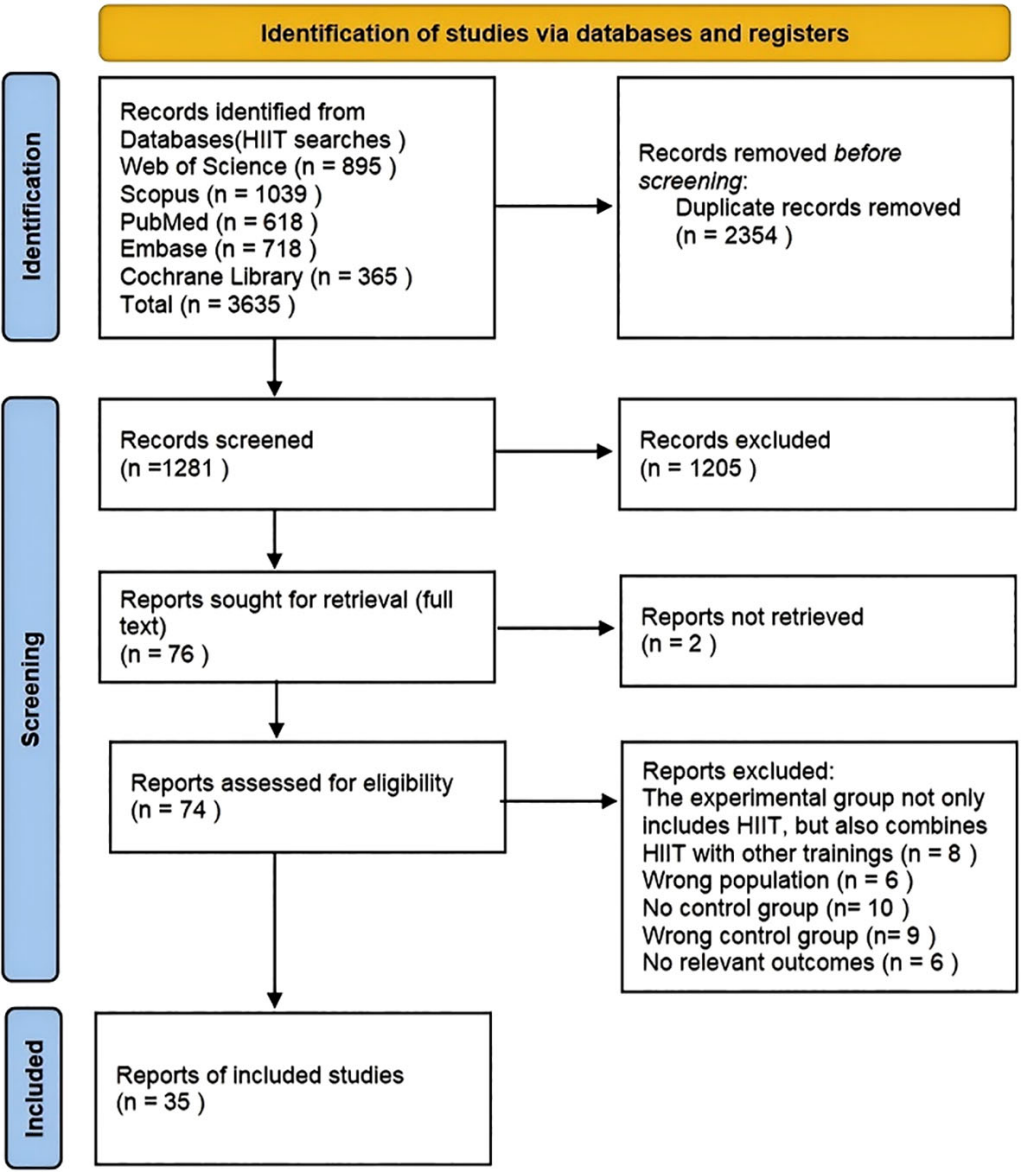
Systematic review search and screening procedure.

The literature screening process is presented in **Figure 1**. In total, 3,635 records were identified. After duplicates were removed, 1,281 records were screened by title and abstract, and 1,205 were excluded at this stage. Then, 76 articles moved to full-text retrieval, but 2 full texts could not be obtained, leaving 74 articles for full-text assessment. After full-text screening, 39 articles were excluded, and 35 studies were finally included in this systematic review and meta-analysis (Aschendorf et al., 2019; Bici and Kasa, 2025; Bilici and Topates, 2025; Buchheit et al., 2010; Chtara et al., 2017; Demirman et al., 2024; Fang et al., 2021; Fernandez-Fernandez et al., 2017; Gökkurt and Kıvrak, 2021; Haghighi et al., 2023; Hammami et al., 2021; He et al., 2025; Helgerud et al., 2001; Hermassi et al., 2018; Howard and Stavrianeas, 2017; Iaia et al., 2017; Jurisic et al., 2023; Kaewkamda et al., 2025; Lambright et al., 2024; Ma et al., 2024; Nascimento et al., 2014; Ojeda-Aravena et al., 2021; Ouergui et al., 2020; Prasad et al., 2025; Qin et al., 2026; Sagat, 2026; Sanchez-Sanchez et al., 2019; Sandbakk et al., 2011; Seo et al., 2019; Sperlich et al., 2011; Tønnessen et al., 2011; Venturelli et al., 2008; Wen et al., 2024; Xu et al., 2024, Xu et al., 2025).

### 3.2 Characteristics of included studies

A total of 35 studies were included, involving 988 trained adolescent athletes. Twenty-one studies included male samples, 10 included female samples, and 4 included mixed samples. The participants’ mean age ranged from approximately 11.0 to 18.4 years. Soccer was the most commonly represented sport (17 studies), followed by basketball and handball (4 studies each), and taekwondo (3 studies). The remaining sports included tennis (2 studies), futsal, karate, cross-country skiing, aerobic gymnastics, and volleyball (1 study each). The competitive level of all participants was classified as Tier 2 or Tier 3, with 19 studies in Tier 2 and 16 in Tier 3. The HIIT types included RST (11 studies), short-interval (10 studies), SIT (5 studies), long-interval (4 studies), and mixed short-/long-interval protocols (4 studies). One study included both RST and short-interval HIIT as two experimental groups. Running-based HIIT was the main HIIT mode (24 studies), whereas sport-specific HIIT was used in 9 studies. In addition, 2 studies included both running-based HIIT and sport-specific HIIT experimental groups. The intervention duration ranged from 4 to 12 weeks, the training frequency ranged from 1 to 4 sessions per week, and the total number of training sessions ranged from 8 to 32. The main outcomes included CMJ and COD (20 studies each), VO_2_max (16 studies), 20m sprint (11 studies), Yo-Yo IR1 (7 studies), RSA (7 studies), and 30–15 IFT (5 studies). More detailed characteristics of the included studies are presented in **Tables 2** and **3**.

**Table 2:** Characteristics of included studies.

Study	Sport	Sex	Age (y)	Sample size	Competitive level	Control intervention	Outcomes
Helgerud et al., 2001	Soccer	M	18.1 ± 0.8	EG: 9 CG: 10	National (Tier 3)	Regular soccer training	VO_2_max, 10 m sprint, 40 m sprint, CMJ
Venturelli et al., 2008	Soccer	M	11.0 ± 0.5	EG: 7 CG: 9	National (Tier 3)	Coordination training	20 m sprint, CMJ, SJ
Buchheit et al., 2010	Soccer	M	14.5 ± 0.5	EG: 7 CG: 8	Developmental (Tier 2)	Strength training	10 m sprint, 30 m sprint, CMJ, RSA
Tønnessen et al., 2011	Soccer	M	16.4 ± 0.9	EG: 10 CG: 10	National (Tier 3)	Regular soccer training	40 m sprint, CMJ, 20 m sprint, RSA
Sperlich et al., 2011	Soccer	M	13.5 ± 0.4	EG: 9 CG: 8	National (Tier 3)	High-volume training	VO_2_max, 20 m sprint, 30 m sprint, 40 m sprint, CMJ, SJ
Sandbakk et al., 2011	Cross-country skiing	Mixed	17.4 ± 0.5	EG: 7 CG: 8	Developmental (Tier 2)	Baseline training	VO_2_max
Nascimento et al., 2014	Futsal	M	16.7 ± 0.5	EG: 8 CG: 6	Developmental (Tier 2)	Regular futsal training	40 m sprint, CMJ
Chtara et al., 2017	Soccer	M	13.6 ± 0.3	EG: 12 CG: 10	National (Tier 3)	Regular soccer training	10 m sprint, 30 m sprint, COD (Zigzag test), RSA
Howard and Stavrianeas, 2017	Soccer	M	EG:15.06 ± 0.77 CG: 14.81 ± 1.22	EG: 16 CG: 16	Developmental (Tier 2)	Traditional endurance running	Yo-Yo IR1, 40-yard dash, CMJ, COD (Illinois test)
Iaia et al., 2017	Soccer	M	17.0 ± 1.0	EG1: 9 EG2: 10 CG: 10	Developmental (Tier 2)	Soccer fitness conditioning	20 m sprint, Yo-Yo IR2
Fernandez-Fernandez et al., 2017	Tennis	M	14.8 ± 0.1	EG: 8 CG: 9	National (Tier 3)	Sport-specific tennis training	VO_2_max, 5 m sprint, 10 m sprint, 20 m sprint, CMJ, 30–15 IFT (VIFT), COD (505 test)
Hermassi et al., 2018	Handball	M	EG: 17.1 ± 0.3 CG: 17.3 ± 0.5	EG: 15 CG: 15	National (Tier 3)	Regular handball training	Yo-Yo IR1, CMJ, SJ, RSA
Aschendorf et al., 2019	Basketball	F	15.1 ± 1.1	EG: 11 CG: 13	National (Tier 3)	Common team-training	Yo-Yo IR1, CMJ, SJ, COD (20 m COD shuttle sprint)
Sanchez-Sanchez et al., 2019	Soccer	M	EG1: 14.7 ± 0.5 EG2: 14.4 ± 0.5 CG: 14.9 ± 0.4	EG: 10 EG: 10 CG: 9	Developmental (Tier 2)	Regular soccer training	Yo-Yo IR1, VO_2_max, COD, RSA
Seo et al., 2019	Taekwondo	M	16.7 ± 0.84	EG1: 12 EG2: 12 EG3: 12 CG: 11	Developmental (Tier 2)	Taekwondo training	VO_2_max, CMJ, COD (T-test)
Ouergui et al., 2020	Taekwondo	Mixed	16.1 ± 1	EG1: 12 EG2: 12 CG: 12	National (Tier 3)	Taekwondo training	VO_2_max, CMJ, COD (T-test)
Gökkurt and Kivrak, 2021	Soccer	M	EG: 18.36 ± 0.51 CG: 17.55 ± 0.69	EG: 11 CG: 10	National (Tier 3)	Tegular soccer training	15 m sprint, 30 m sprint, COD (Pro-agility test)
Ojeda-Aravena et al., 2021	Karate	Mixed	EG: 16.1 ± 1.12 CG: 14.5 ± 2.0	EG: 5 CG: 5	National (Tier 3)	Regular karate training	CMJ, SJ, COD (T-test)
Fang et al., 2021	Soccer	M	EG: 15.7 ± 0.8 CG: 15.8 ± 0.7	EG: 27 CG: 29	Developmental (Tier 2)	Moderate-intensity continuous training	VO_2_max
Hammami et al., 2021	Handball	M	EG: 16.6 ± 0.5 CG: 16.5 ± 0.8	EG: 17 CG: 15	National (Tier 3)	Regular handball training	CMJ, SJ, 5 m sprint, 10 m sprint, 20 m sprint, 30 m sprint, VO_2_max, COD (Illinois test), RSA
Jurisic et al., 2023	Handball	F	EG: 16.13 ± 0.91 CG: 16.00 ± 0.93	EG: 15 CG: 15	Developmental (Tier 2)	Regular handball training	COD (T-test)
Haghighi et al., 2023	Basketball	F	EG: 15.1 ± 1.6 CG: 15.1 ± 1.8	EG: 8 CG: 8	National (Tier 3)	Regular basketball training	20 m sprint, COD
Demirman et al., 2024	Handball	F	EG: 16.80 ± 1.87 CG: 16.70 ± 1.82	EG: 10 CG: 10	Developmental (Tier 2)	Regular handball training	COD (T-test), 20 m sprint, 30–15 IFT (VIFT)
Lambright et al., 2024	Soccer	F	EG: 16.06 ± 1.48 CG: 16.25 ± 1.83	EG: 16 CG: 16	Developmental (Tier 2)	Resistance training	36.5 m sprint, CMJ, COD (Illinois test)
Ma et al., 2024	Aerobic gymnastics	F	EG: 16.2 ± 1.3 CG: 16.2 ± 1.4	EG: 24 CG: 25	Developmental (Tier 2)	Regular aerobic gymnastics training	CMJ, 20 m multistage fitness test
Xu et al., 2024	Basketball	M	EG: 16.1 ± 0.9 CG: 16.2 ± 0.6	EG: 11 CG: 10	National (Tier 3)	Regular basketball training	VO_2_max, 20 m sprint, COD (T-test), CMJ, SJ
Wen et al., 2024	Soccer	F	EG: 17.1 ± 0.8 CG: 17.0 ± 1.1	EG: 16 CG: 16	Developmental (Tier 2)	Regular soccer training	CMJ, COD (505 test), 30–15 IFT (VIFT)
Bici and Kasa, 2025	Soccer	M	16.2 ± 0.4	EG: 17 CG: 27	Developmental (Tier 2)	Regular soccer training	VO_2_max, Yo-Yo IR1
Bilici and Topates, 2025	Volleyball	F	EG: 16.00 ± 1.05 CG: 16.64 ± 1.29	EG: 10 CG: 11	Developmental (Tier 2)	Regular volleyball training	CMJ, 20 m sprint, Yo-Yo IR1, VO_2_max, COD (Pro-agility test)
He et al., 2025	Basketball	M	16.5 ± 0.7	EG1: 12 EG2: 12 CG: 12	Developmental (Tier 2)	Regular basketball training	30–15 IFT (VIFT)
Kaewkamda et al., 2025	Soccer	F	14-18	EG: 15 CG: 15	Developmental (Tier 2)	Regular soccer training	Yo-Yo IR1, VO_2_max
Prasad et al., 2025	Soccer	F	EG: 15.11 ± 0.92 CG: 15.77 ± 0.97	EG: 9 CG: 9	Developmental (Tier 2)	Regular soccer training	COD (Illinois test), 50 m sprint
Xu et al., 2025	Taekwondo	Mixed	EG1: 16.80 ± 1.4 EG2: 16.60 ± 1.43 CG: 17.60 ± 1.07	EG1: 10 EG2: 10 CG: 10	National (Tier 3)	Regular karate training	VO_2_max, COD (Illinois test)
Qin et al., 2026	Tennis	M	16.8 ± 0.8	EG1: 12 EG2: 12 EG3: 12 CG: 12	Developmental (Tier 2)	Regular tennis training	VO_2_max, 30–15 IFT (VIFT)
Sagat, 2026	Soccer	M	EG1: 16.38 ± 0.24 EG2: 16.46 ± 0.20 CG: 16.41 ± 0.19	EG1: 20 EG2: 20 CG: 20	National (Tier 3)	Regular soccer training	VO_2_max, 5 m sprint, 10 m sprint, 20 m sprint, CMJ, SJ, COD (505), RSA

M, male; FM, female; Mixed, male and female; VO_2_max, maximal oxygen uptake; Yo-Yo IR1, Yo-Yo Intermittent Recovery Test Level 1; Yo-Yo IR2, Yo-Yo Intermittent Recovery Test Level 2; 30–15 IFT, 30–15 Intermittent Fitness Test; VIFT, final velocity reached in the 30–15 Intermittent Fitness Test; CMJ, countermovement jump; SJ, squat jump; COD, change-of-direction; RSA, repeated sprint ability.

**Table 3:** Characteristics of HIIT interventions in the included studies.

Study	Sport	HIIT type	HIIT mode	Season	Duration (weeks)	Frequency (sessions)	Total sessions (n)	Sets	Reps	Work duration	Work intensity	Recovery between sets (s)	Recovery between reps (time)	Type of recovery (intensity)
Helgerud et al., 2001	Soccer	Long-interval	Running-based HIIT	Pre-season	8	2	16	1	4	4 min	90–95% HRmax	NR	3 min	Active recovery jog at 50–60% HRmax
Venturelli et al., 2008	Soccer	RST	Running-based HIIT	In-season	12	2	24	NR	20 total	NR	Maximal	NR	60–90 s	Passive recovery
Buchheit et al., 2010	Soccer	RST	Running-based HIIT	NR	10	1	10	2-3	5-6	15 or 20 m sprint	Maximal	NR	14s or 23s	Passive recovery or Active recovery
Tønnessen et al., 2011	Soccer	RST	Running-based HIIT	Pre-season	10	1	10	2–5	4–5	40 m sprint	Maximal	600 s	90–120 s	NR
Sperlich et al., 2011	Soccer	Short-interval / Long-interval	Running-based HIIT	Pre-season	5	3-4	13	1	4-12	30 s 1 min 4min 200m, 400m, 800m sprint	90–95% HRmax	30–180 s	NA	Active recovery at 50–60% HRmax
Sandbakk et al., 2011	Cross-country skiing	Long-interval	Sport-specific HIIT	Pre-season	8	1	8	NR	NR	5–10 min	85–92% HRmax	NR	NR	NR
Nascimento et al., 2014	Futsal	RST	Running-based HIIT	In-season	4	2	8	3	6	40 m sprint	Maximal	240 s	20s	Passive recovery
Chtara et al., 2017	Soccer	RST	Running-based HIIT	In-season	6	2	12	2–4	5–6	20–30 m shuttle sprints with 180° turns	Maximal (100%)	240 s	20 s	Passive recovery
Howard and Stavrianeas, 2017	Soccer	SIT	Running-based HIIT	In-season	10	3	30	1	4 (wk1–2), 5 (wk3–4), 6 (wk5–10)	30 s	All-out	NR	270 s	Active recovery
Iaia et al., 2017	Soccer	RST	Running-based HIIT	In-season	5	2	8	1-3	6	5 s (30 m sprint)	Maximal	120 s	15 s (EG1) 30 s (EG2)	Passive recovery
Fernandez-Fernandez et al., 2017	Tennis	Short-interval	Running-based HIIT	Pre-season	8	1	8	2	15-22	15s	90-95% VIFT	180 s	15 s	Passive recovery
Hermassi et al., 2018	Handball	Short-interval	Running-based HIIT	In-season	7	2	14	1-5	5 or 10	10 or 20 s	110–130% MAS	180 s	work: rest 1:1	NR
Aschendorf et al., 2019	Basketball	Short-interval/Long-interval	Sport-specific HIIT	In-season	5	2	10	A: 1 B: 2	A: 4 B: 15	A: 4 min B: 30 s	90–95% HRmax	A: NA B: 180 s	A: 180 s B: 15 s	A/B: NR
Sanchez-Sanchez et al., 2019	Soccer	RST	Running-based HIIT	NR	8	2	16	3	10	18 m sprint	Maximal	240 s	NR	Active recovery
Seo et al., 2019	Taekwondo	SIT	Sport-specific HIIT	NR	4	Wk1, 3: 2 Wk2, 4: 3	10	1	Wk1–2: 6 Wk3–4:8	30 s	90–100% HRmax	NA	EG1: 60 s EG2: 120 s EG3: 240 s	Active recovery walking
Ouergui et al., 2020	Taekwondo	EG1: RST EG2: Short-interval	EG1: Running-based HIIT EG2: Sport-specific HIIT	NR	4	2	8	3 (wk1) 4 (wk2) 5 (wk3) 6(wk4)	10	EG1: 35 m sprint EG2: 6 s	Maximal	180 s	10 s	Passive recovery
Gökkurt and Kivrak, 2021	Soccer	Long-interval	Running-based HIIT	NR	8	3	24	3	NR	90 s jogging	90% HRmax	120 s	90s	Walking (rest)
Ojeda-Aravena et al., 2021	Karate	Short-interval	Sport-specific HIIT	Pre-season	4	Wk1-2: 2 Wk3-4: 3	10	3	15	4 s	Maximal	180 s (wk1–2) 180s (wk3–4)	8 s	Active recovery
Fang et al., 2021	Soccer	SIT	Running-based HIIT	NR	4	3	12	2	6	30 s	Maximal	600 s	120 s	Active recovery
Hammami et al., 2021	Handball	RST	Running-based HIIT	In-season	8	2	16	4	8	5s	130% aerobic maximum speed	180 s-300 s	10 s	NR
Jurisic et al., 2023	Handball	Short-interval	Running-based HIIT	Pre-season	8	4	32	2	NR	15 s	90–95% MAS (progressed 90→92→95%)	180 s	15 s	Active recovery
Demirman et al., 2024	Handball	Short-interval	Sport-specific HIIT	In-season	8	3	24	3	4	20s,30s,60 s	Maximal	30s	30 s	NR
Haghighi et al., 2023	Basketball	Short-interval	Sport-specific HIIT	Pre-season	6	2	12	1 (wk1–4) 2 (wk5–6)	7–8	30–45 s	Maximal-effort	120 s	15–20 s	NR
Lambright et al., 2024	Soccer	SIT	Sport-specific HIIT	Pre-season	8	3	24	3,5-6	NR	20 s (wk1–2), 30 s (wk3–5), 40 s (wk6–8)	All-out	NR	10–20 s	NR
Ma et al., 2024	Aerobic gymnastics	Short-interval	Running-based HIIT	NR	8	2	16	4 (wk1–4) 5 (wk5–8)	NA	30 s (wk1–2,5–6), 40 s (wk3–4,7–8)	110% MAS (30 s), 105% MAS (40 s)	30 s	NR	NR
Xu et al., 2024	Basketball	RST	Running-based HIIT	Off-season	6	3	18	3	12	5 s	Sprint	120 s	20 s	NR
Wen et al., 2024	Soccer	Short-interval/Long-interval	Running-based HIIT	Pre-season	8	2	16	4	2 (wk1–4), 4(wk5–8)	2 min (wk1–4) 4 min (wk5–8)	90% VIFT (wk1–4) and 85% VIFT (wk5–8)	15 s (wk1–4), 180s (wk5–8),	180 s	NR
Bici and Kasa, 2025	Soccer	SIT	Running-based HIIT	In-season	8	2	16	1	7	20 s	All-out	NA	100 s	Passive recovery
Bilici and Topates, 2025	Volleyball	Short-interval	Sport-specific HIIT	In-season	12	2	24	4 (wk1–4) 5 (wk5–8) 6 (wk9–12)	NR	30 s	85–95% HRmax	60 s	30 s	Active recovery
He et al., 2025	Basketball	Long-interval	Running-based HIIT	In-season	6	2	12	3–5	NR	3–4 min	85–95% VIFT	120 s	NR	Active recovery at 55% VIFT
Kaewkamda et al., 2025	Soccer	RST	Running-based HIIT	NR	6	2	12	1	6	20 m sprint	Maximal	NR	180 s	NR
Prasad et al., 2025	Soccer	Short-interval / Long-interval	Running-based HIIT	In-season	8	3	24	NR	Wk1–2: 10–12; Wk3–4: 6; Wk5–6: 8; Wk7–8: 10–12	Wk1–2: 30s; Wk3–4: 200 m; Wk5–6: 30 s; Wk7–8: 20 s	85–95% HRmax	NR	Wk1–2: 30 s Wk5–6: 15 s Wk7–8: 10 s	Passive recovery or jog/walk recovery
Xu et al., 2025	Taekwondo	Short-interval	Sport-specific HIIT	NR	8	3	24	3	4	20 s	Maximal	60 s	10 s	NR
Qin et al., 2026	Tennis	Short-interval	EG1: Sport-specific HIIT EG2: Sport-specific HIIT EG3: Running-based HIIT	Pre-season	6	2	12	EG1:2 EG2: 2 EG3: 1	EG1 and EG2: 6-14 EG3: 12-24	30–40 s	EG1 and EG2: Maximal EG3: 85–95% VIFT	EG1: 180 s EG2:180 s EG3: NA	30 s	Passive recovery
Sagat, 2026	Soccer	RST	Running-based HIIT	Pre-season	12	2	24	3	Wk1–4: 10 Wk5–8: 15 Wk9–12: 20	EG1: 15 m shuttle sprint with 180 °CoD (<10 s) EG2: 31 m linear sprint (<10 s)	Maximal	240 s	<30 s	Low-intensity running

HIIT, high-intensity interval training; SIT, sprint interval training; RST, repeated sprint training; HRmax, maximal heart rate; MAS, maximal aerobic speed; VIFT, final velocity reached in the 30–15 Intermittent Fitness Test; NR, not reported; NA, not applicable.

### 3.3 Risk of bias assessment

**Figure 2:**
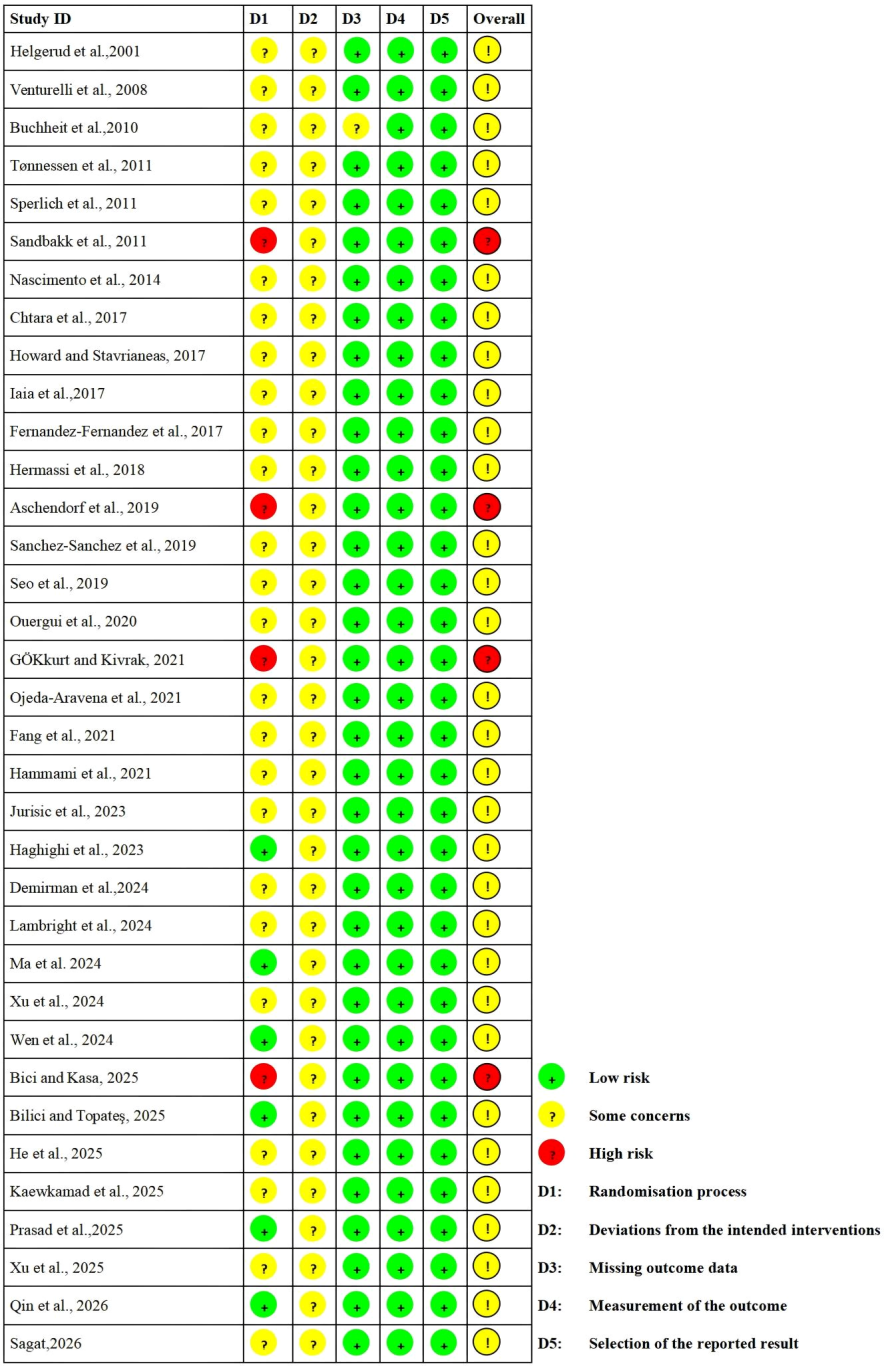
RoB 2 assessments.

**Figure 3:**
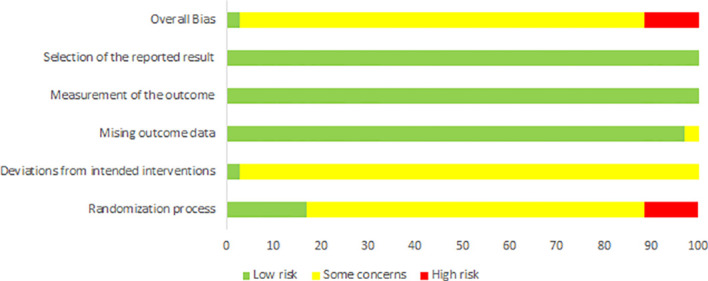
Risk of overall bias.

The results of the risk of bias assessment for the included studies are shown in **Figures 2** and **3**. Of the 35 studies, 31 were judged to have some concerns regarding the overall risk of bias, whereas 4 were judged to be at high risk. For the randomization process domain, 6 studies were rated as low risk, 25 as some concerns, and 4 as high risk, mainly due to unclear reporting of random sequence generation or the randomization process, and insufficient information on allocation concealment. For deviations from intended interventions, all 35 studies were rated as having some concerns, likely mainly due to the difficulty of blinding in exercise training studies. For missing outcome data, 34 studies were rated as low risk, and 1 study was rated as some concerns. For measurement of the outcome and selection of the reported result, all studies were rated as low risk. Overall, the risk of bias was mainly concentrated in the domains of the randomization process and deviations from intended interventions.

### 3.4 Meta-analysis results

The pooled meta-analytic results for all outcomes are presented in **Table 4**, and the corresponding forest plots are shown in **Figures 4**–**11**.

**Table 4:** Meta-analytic effects of HIIT on physical fitness in trained adolescent athletes.

Outcome	K, n	SMD (95%CI)	P (Overall Effect)	I^2^ (%)	RW (%)	Egger’s test (p)
VO_2_max	16,522	0.65 (0.46 to 0.83)	<0.001	0	2.10–12.40	0.707
Field-based intermittent endurance test performance	9,288	0.65 (0.07 to 1.23)	0.028	79.4	7.85–10.47	NR
30–15 Intermittent Fitness Test (VIFT)	5,153	1.13 (0.63 to 1.63)	<0.001	42.5	9.61–18.30	NR
Countermovement jump	20,543	0.44 (0.13 to 0.74)	0.004	62.7	2.80-5.59	0.194
≤10 m sprint	6,165	-0.79 (-1.26 to -0.32)	0.001	50.1	12.35–15.99	NR
≥20 m sprint	18,423	-0.28 (-0.47 to -0.08)	0.005	0	3.33–7.78	0.785
Change-of-direction (COD)	20,550	-0.54 (-0.72 to -0.37)	<0.001	0	1.87–6.56	0.691
Repeated sprint ability (RSA)	7,208	-0.70 (-1.02 to -0.39)	<0.001	12.7	6.75–14.24	NR

K, Data denote the number of studies that provided data for the analysis; n, The total number of trained adolescent athletes included in the analysis, respectively; NR, Less than 10 studies were included, and publication bias was not evaluated.

**Figure 4:**
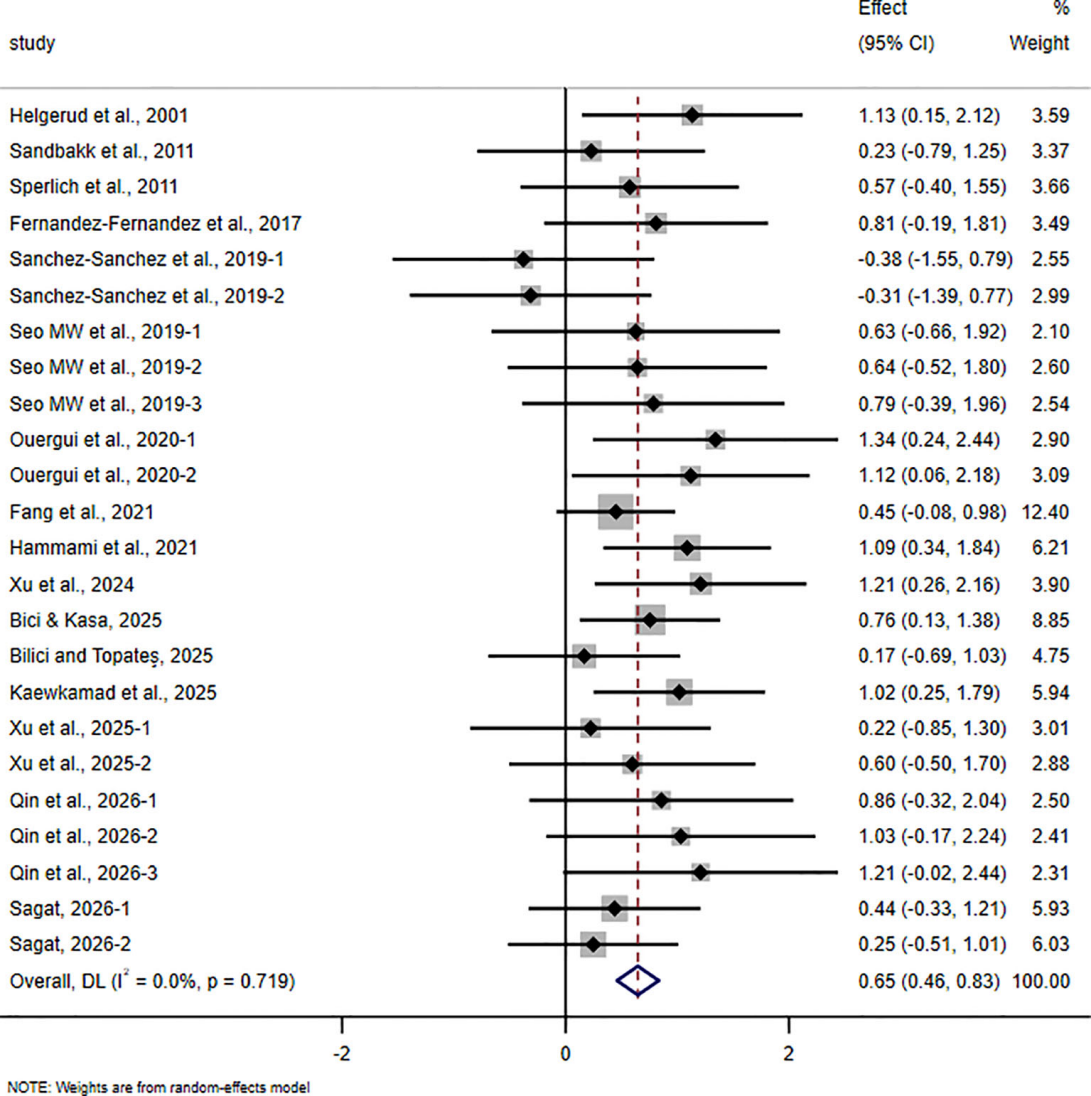
Forest plot of the effect of HIIT on VO_2_max in trained adolescent athletes.

**Figure 5:**
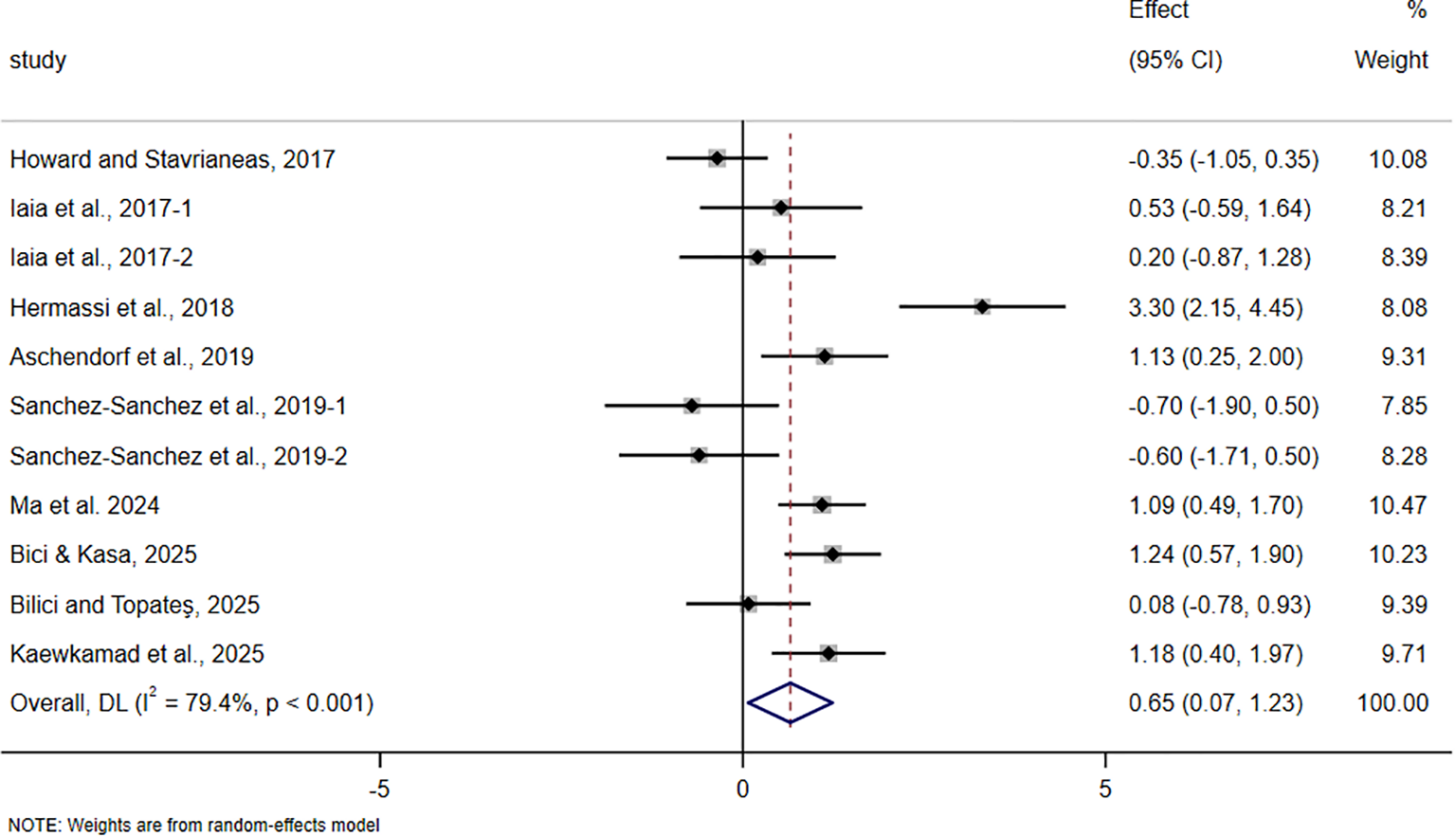
Forest plot of the effect of HIIT on field-based intermittent endurance test performance in trained adolescent athletes.

**Figure 6:**
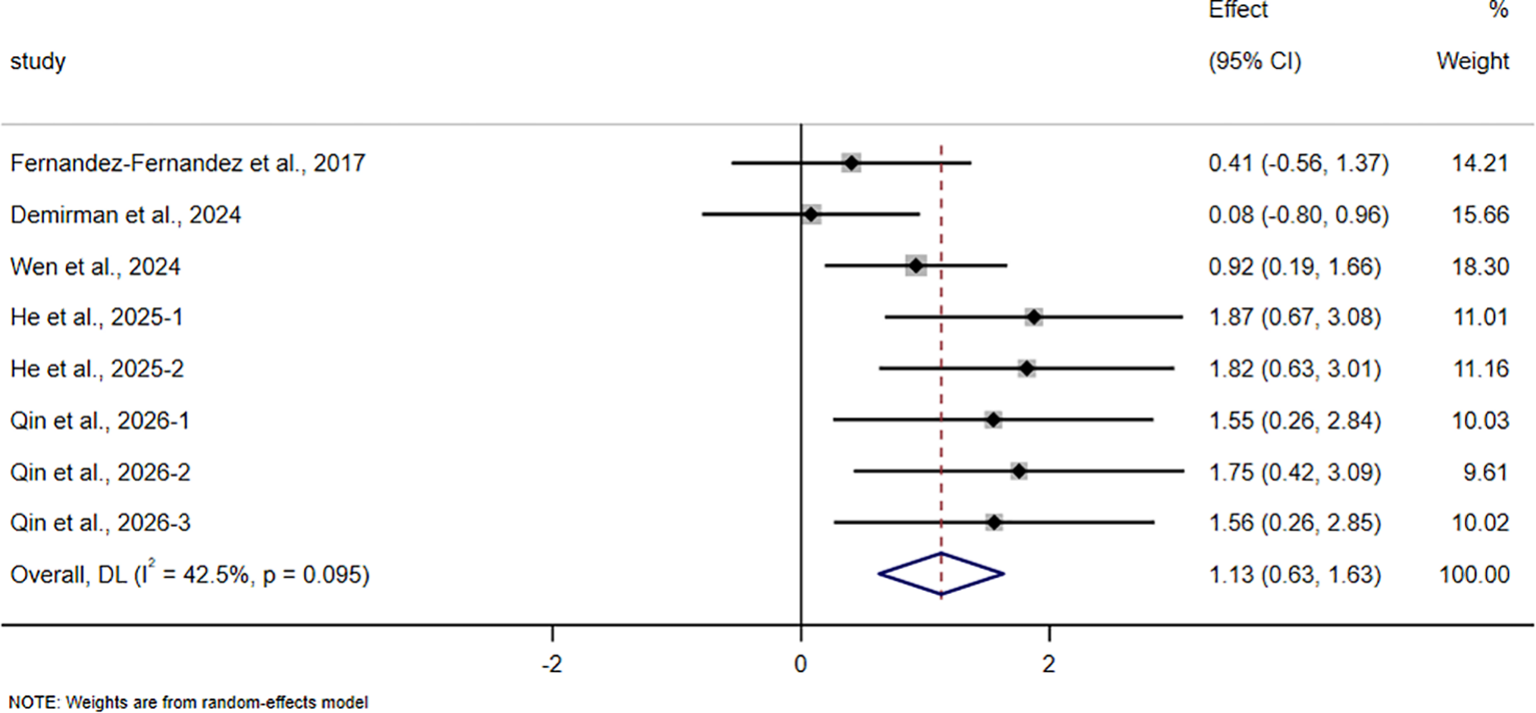
Forest plot of the effect of HIIT on VIFT in trained adolescent athletes.

**Figure 7:**
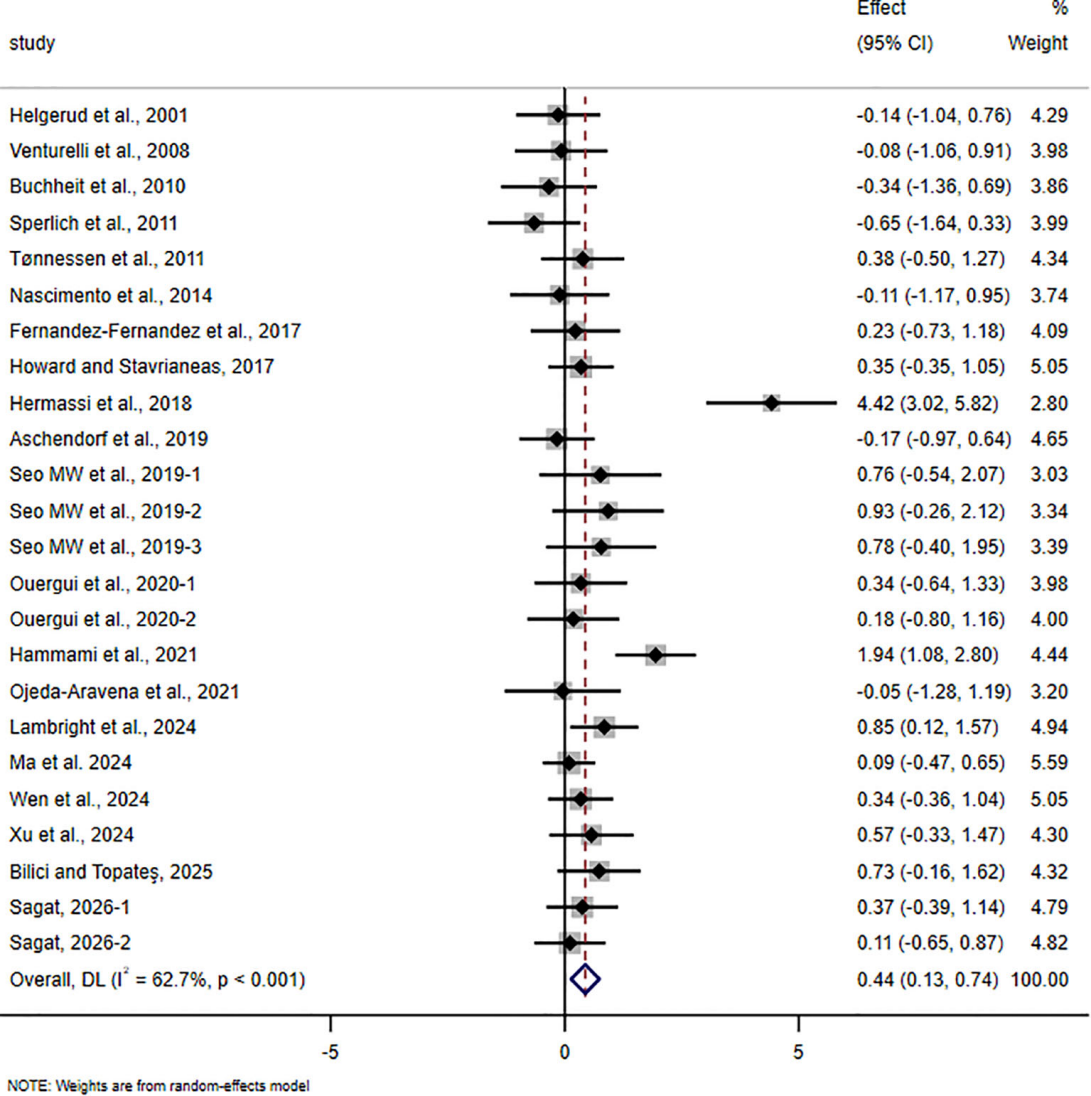
Forest plot of the effect of HIIT on CMJ in trained adolescent athletes.

**Figure 8:**
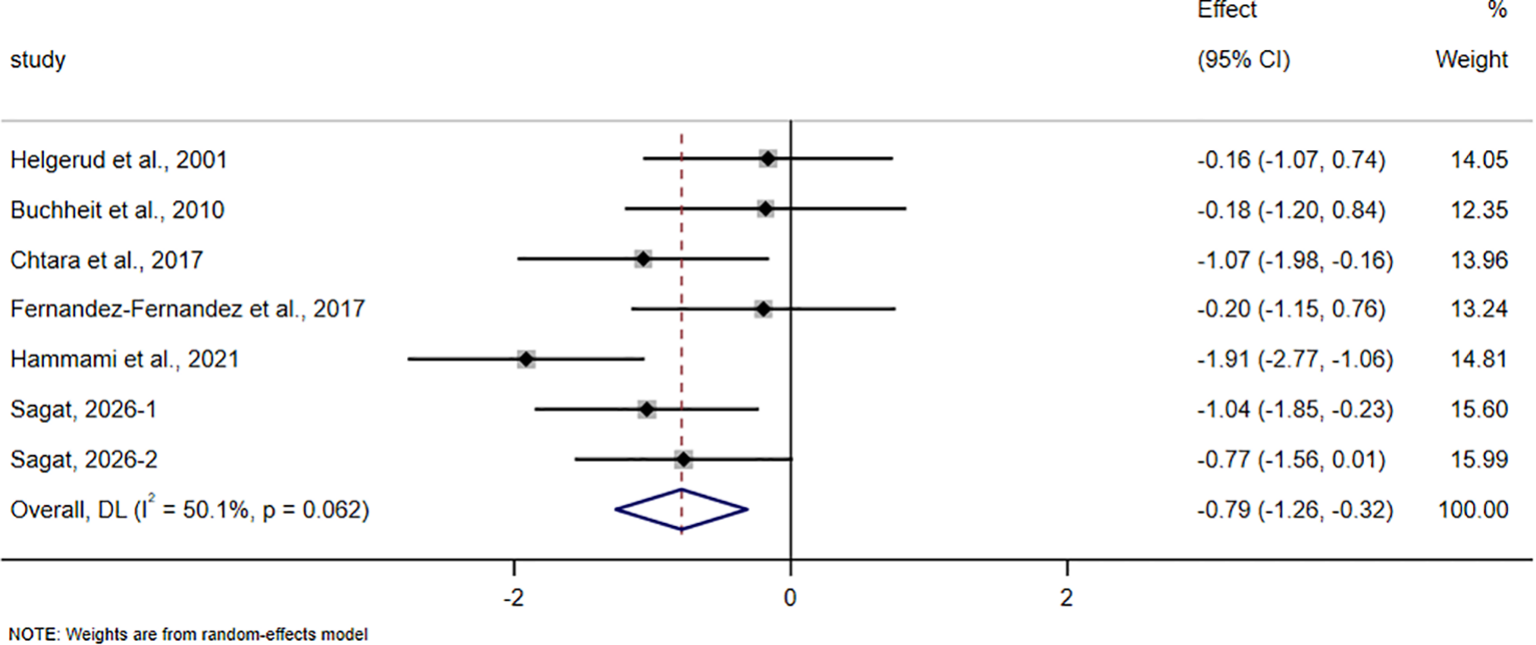
Forest plot of the effect of HIIT on ≤ 10 m sprint in trained adolescent athletes.

**Figure 9:**
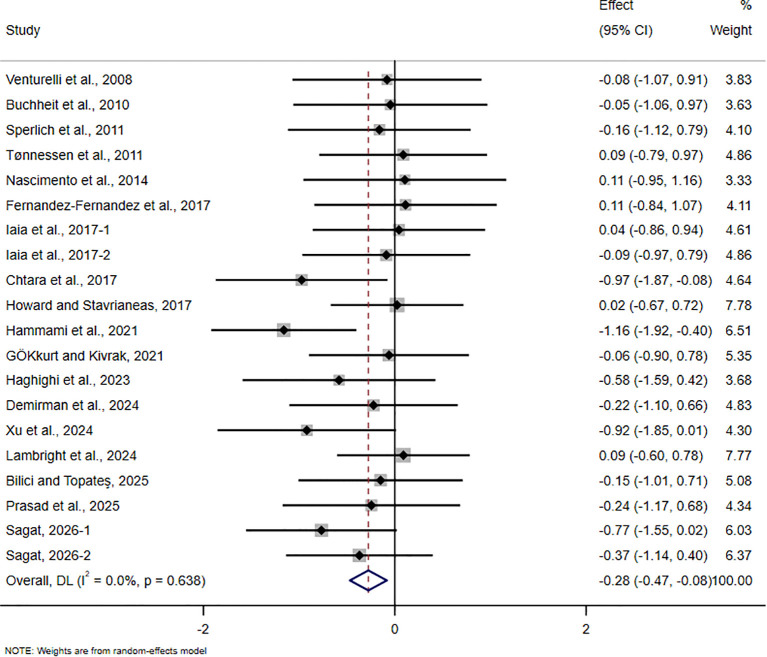
Forest plot of the effect of HIIT on ≥ 20 m sprint in trained adolescent athletes.

**Figure 10:**
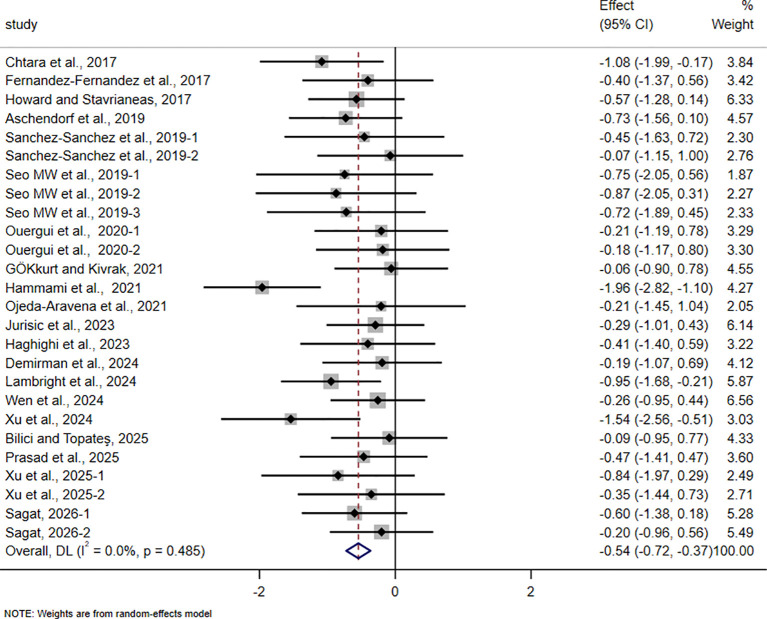
Forest plot of the effect of HIIT on COD in trained adolescent athletes.

**Figure 11:**
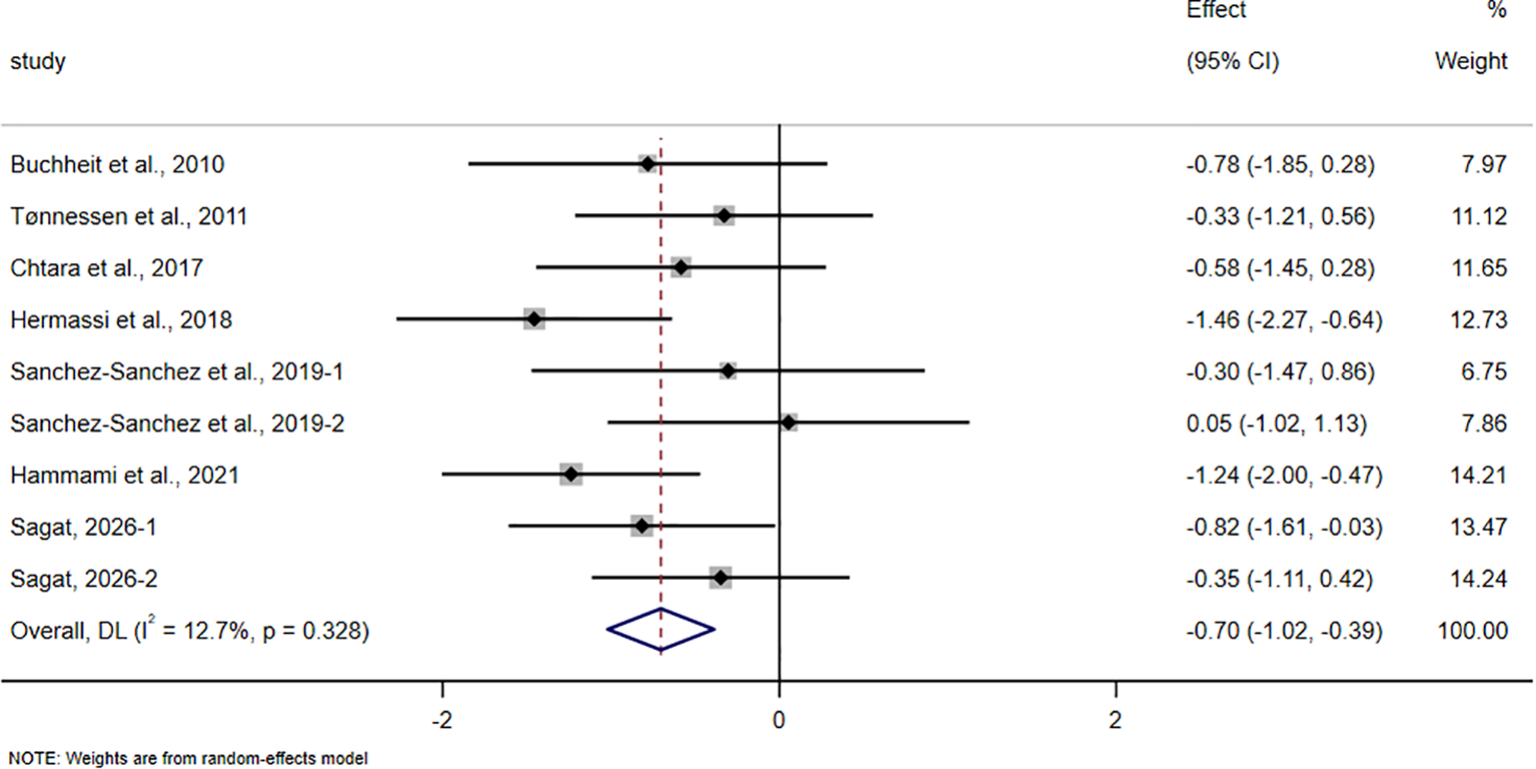
Forest plot of the effect of HIIT on RSA in trained adolescent athletes.

#### 3.4.1 Impact of HIIT on cardiorespiratory fitness

For VO_2_max, 16 studies involving 522 participants were included. The pooled analysis showed that HIIT-based interventions significantly improved VO_2_max in trained adolescent athletes (SMD = 0.65, 95% CI 0.46 to 0.83, p < 0.001), with a moderate effect size and no between-study heterogeneity (I² = 0%).

For field-based intermittent endurance test performance, including Yo-Yo IR1, Yo-Yo IR2, and the 20m multistage fitness test, 9 studies involving 288 participants were included. The pooled analysis showed that HIIT-based interventions significantly improved this outcome (SMD = 0.65, 95% CI 0.07 to 1.23, p = 0.028), with a moderate effect size, although between-study heterogeneity was very high (I² = 79.4%).

For the VIFT, 5 studies involving 153 participants were included. The pooled analysis showed that HIIT-based interventions significantly improved performance in this test (SMD = 1.13, 95% CI 0.63 to 1.63, p < 0.001), with a moderate magnitude of improvement and moderate between-study heterogeneity (I² = 42.5%).

#### 3.4.2 Impact of HIIT on jump performance

For CMJ, 20 studies involving 543 participants were included. The pooled analysis showed that HIIT-based interventions significantly improved CMJ in trained adolescent athletes (SMD = 0.44, 95% CI 0.13 to 0.74, p = 0.004), although the magnitude of improvement was small and between-study heterogeneity was high (I² = 62.7%).

#### 3.4.3 Impact of HIIT on sprint performance

Sprint outcomes included ≤10m sprint and ≥20m sprint. For the ≤10m sprint, 6 studies involving 165 participants were included. The pooled analysis showed that HIIT-based interventions significantly improved ≤ 10 m sprint performance in trained adolescent athletes (SMD = -0.79, 95% CI -1.26 to -0.32, p = 0.001), with a moderate effect size and high between-study heterogeneity (I² = 50.1%).

For the ≥20m sprint, 18 studies involving 423 participants were included. The pooled analysis showed that HIIT-based interventions significantly improved sprint performance by ≥20m in trained adolescent athletes (SMD = -0.28, 95% CI -0.47 to -0.08, p = 0.005). However, the magnitude of improvement was small, and no between-study heterogeneity was observed (I² = 0%).

#### 3.4.4 Impact of HIIT on COD

For COD, 20 studies involving 550 participants were included. The pooled analysis showed that HIIT-based interventions significantly improved COD in trained adolescent athletes (SMD = -0.54, 95% CI -0.72 to -0.37, p < 0.001). However, the magnitude of improvement was small, and no between-study heterogeneity was observed (I² = 0%).

#### 3.4.5 Impact of HIIT on RSA

In the RSA analysis, all studies used repeated sprint training (RST) protocols. A total of 7 studies involving 208 adolescent athletes were included. The pooled results showed that HIIT-based interventions, primarily based on RST, significantly improved RSA (SMD = -0.70, 95% CI -1.02 to -0.39, p < 0.001), with a moderate effect size and low between-study heterogeneity (I² = 12.7%).

### 3.5 Subgroup analyses and meta-regression

The subgroup analysis results are presented in **Supplementary Materials**, **Supplementary Table 2**-**S7**.

Overall, no significant between-subgroup differences were observed for most outcomes. For VO_2_max, the between-subgroup differences for age, competitive level, intervention duration, training frequency, total number of training sessions, HIIT type, and HIIT mode did not reach statistical significance (all p > 0.05). Likewise, no significant between-subgroup differences were observed for CMJ, COD, or RSA. Given the generally small number of studies within many subgroup categories, these analyses should be interpreted as exploratory and underpowered, and should not be considered definitive evidence of moderator effects.

For field-based intermittent endurance test performance, age showed the only significant between-subgroup difference (p = 0.033). Specifically, the effect of HIIT-based interventions was significant in athletes aged ≥ 16 years (SMD = 1.07, 95% CI 0.43 to 1.70, p = 0.001), whereas no significant improvement was observed in the <16 years subgroup (SMD = -0.09, 95% CI -0.95 to 0.76, p = 0.837).

For the ≥20m sprint, the competitive level showed the only significant between-subgroup difference (p = 0.034). Specifically, a statistically significant improvement was observed in Tier 3 athletes (SMD = -0.47, 95% CI -0.75 to -0.20, p < 0.001), whereas no significant effect was observed in Tier 2 athletes (SMD = -0.04, 95% CI -0.33 to 0.24, p = 0.783).

The exploratory univariable meta-regression for CMJ did not identify significant associations with age, intervention duration, or training frequency. Detailed results are presented in **Supplementary Table 8**.

### 3.6 Sensitivity analysis

The leave-one-out sensitivity analysis showed that, after excluding each study in turn, the direction and statistical significance of the pooled effects for most outcomes did not change materially, suggesting that the main results were generally robust. Among these outcomes, heterogeneity decreased more markedly for CMJ, VIFT, and ≤10m sprint after specific studies were excluded. Specifically, for CMJ, exclusion of Hermassi et al., 2018 reduced I² from 62.7% to 24.7%; for VIFT, exclusion of Demirman et al., 2024 reduced I² from 42.5% to 8.8%; and for ≤10m sprint, exclusion of Venturelli et al., 2008 reduced I² from 50.1% to 0%. These findings suggest that certain individual studies may have contributed to heterogeneity in these outcomes. However, the direction and statistical significance of the pooled effects did not change materially after exclusion of these studies, and the remaining between-study variability may still reflect differences in participant characteristics, training protocols, and testing methods. Detailed results are provided in the **Supplementary Materials** (**Supplementary Figure 1**-**S8**).

### 3.7 Publication bias assessment

The results of the publication bias assessment are presented in **Table 4**. For outcomes that included at least 10 studies, Egger’s test showed no evident publication bias for VO_2_max (p = 0.707), CMJ (p = 0.194), ≥ 20m sprint (p = 0.785), or COD (p = 0.691). For outcomes with fewer than 10 studies, including field-based intermittent endurance test performance, the VIFT, ≤10m sprint, and RSA, publication bias was not tested due to the limited number of studies.

### 3.8 GRADE assessment of the certainty of evidence

The GRADE assessment results are shown in **Table 5**. The certainty of evidence was rated as moderate for VO_2_max, ≥20m sprint, and COD, low for VIFT, CMJ, ≤10m sprint, and RSA, and very low for field-based intermittent endurance test performance. All outcomes were downgraded for risk of bias, mainly because most included studies had methodological limitations. Some outcomes were further downgraded for inconsistency due to substantial heterogeneity across studies. Other outcomes were also downgraded for imprecision, mainly because the total sample size was relatively small or the confidence intervals of the pooled effect estimates were wide.

**Table 5:** GRADE analysis.

Outcome measures	Number of studies (participants)	Risk of bias	Inconsistency	Indirectness	Imprecision	Other considerations	Quality
VO_2_max	16 (522)	Serious^a^	Not serious	Not serious	Not serious	None	⨁⨁⨁◯ Moderate
Field-based intermittent endurance test performance	9 (288)	Serious^a^	Serious^b^	Not serious	Serious^c^	None^d^	⨁◯◯◯ Very low
30–15 Intermittent Fitness Test (VIFT)	5 (153)	Serious^a^	Not serious	Not serious	Serious^c^	None^d^	⨁⨁◯◯ Low
Countermovement jump (CMJ)	20 (543)	Serious^a^	Serious^b^	Not serious	Not serious	None	⨁⨁◯◯ Low
≤10 m sprint	6 (165)	Serious^a^	Not serious	Not serious	Serious^c^	None^d^	⨁⨁◯◯ Low
≥20 m sprint	18 (423)	Serious^a^	Not serious	Not serious	Not serious	None	⨁⨁⨁◯ Moderate
Change-of-direction (COD)	20 (550)	Serious^a^	Not serious	Not serious	Not serious	None	⨁⨁⨁◯ Moderate
Repeated sprint ability (RSA)	7 (208)	Serious^a^	Not serious	Not serious	Serious^c^	None^d^	⨁⨁◯◯ Low

^a^ : Most included studies had some methodological limitations. ^b^: Substantial heterogeneity was observed across studies for this outcome. ^c^: The total sample size was relatively small and/or the confidence interval around the pooled estimate was relatively wide, which reduced confidence in the effect estimate. ^d^: Publication bias was not downgraded because outcomes with fewer than 10 studies were not formally assessed.

## 4 Discussion

This study examined the effects of HIIT-based interventions on physical fitness in trained adolescent athletes through a systematic review and meta-analysis. Compared with non-HIIT comparator conditions, pooled analyses suggested beneficial effects of HIIT-based interventions across several outcomes in trained adolescent athletes, although confidence in these effects was not uniform across outcomes. The improvement reached a moderate magnitude for VO_2_max, field-based intermittent endurance test performance, the VIFT, and ≤10m sprint, while the gains in CMJ, ≥20m sprint, and COD were relatively small. Sensitivity analyses further showed that removing studies one at a time did not materially change the direction or statistical significance of the effects for most outcomes, indicating that the pooled estimates were generally stable in leave-one-out analyses. Even so, the certainty of evidence varied across outcomes, and ratings of low or very low should still be interpreted with caution.

### 4.1 Cardiorespiratory fitness

HIIT-based interventions significantly improved VO_2_max, field-based intermittent endurance test performance, and the VIFT in trained adolescent athletes. VO_2_max reflects the overall functional status of the cardiorespiratory system and muscular oxidative function and is an important indicator of endurance performance (Bassett, 2000; Poole and Jones, 2017). HIIT-based interventions may enhance VO_2_max through repeated exposure to exercise intensities at or near VO_2_max, which may induce both central and peripheral adaptations (Buchheit and Laursen, 2013; Midgley et al., 2006). These adaptations may include greater stroke volume and cardiac output, together with improvements in skeletal muscle oxidative capacity, mitochondrial enzyme activity, and capillary density (Jacobs et al., 2013; MacInnis and Gibala, 2017; Rosenblat et al., 2022).

In contrast, field-based intermittent endurance test performance and VIFT more strongly reflect the athlete’s ability to maintain exercise output and recover rapidly during repeated high-intensity running (Bangsbo et al., 2008; Buchheit, 2008). Accordingly, the improvements induced by HIIT-based interventions may be related to enhanced phosphagen recovery, better lactate transport and clearance, and improved muscle buffering capacity, which may support faster recovery between efforts and help athletes maintain performance during high-intensity intermittent exercise (Edge et al., 2006; Germano et al., 2015; Mendez-Villanueva et al., 2012; Wiewelhove et al., 2015).

Subgroup analyses showed that the effect of HIIT-based interventions on VO_2_max was generally consistent across age, competitive level, intervention duration, training frequency, total number of training sessions, HIIT type, and HIIT mode, with no significant between-subgroup differences. In contrast, for field-based intermittent endurance test performance, a significant between-subgroup difference was observed only in the age-subgroup analysis, with the improvement mainly observed in the ≥ 16 years subgroup. This finding only suggests that age or maturity status may be associated with the magnitude of adaptation in intermittent endurance performance. This may be related to greater biological maturity, maturity-related physiological and neuromuscular development, and longer training exposure in older adolescent athletes (Beunen and Malina, 2008; Bidaurrazaga-Letona et al., 2019; Wright et al., 2016). However, the Yo-Yo IR1, Yo-Yo IR2, and the 20m multistage fitness test, included under field-based intermittent endurance test performance, are not fully equivalent in test structure, movement patterns, or physiological demands (Bangsbo et al., 2008; Leger et al., 1988). Therefore, given the lack of test uniformity, high heterogeneity, and limited certainty of the evidence, these findings should be interpreted with caution.

### 4.2 Jump performance

HIIT-based interventions significantly improved CMJ performance in trained adolescent athletes, although the magnitude of improvement was relatively small. This finding differs from previous systematic reviews that included both adults and adolescents (Qi et al., 2026), and this difference may be related to the training adaptations of adolescent athletes. Adolescence represents a sensitive period for neuromuscular development, during which the stretch-shortening cycle, movement coordination, and rapid force production show considerable plasticity (Radnor et al., 2018).

Particularly in HIIT-based interventions, characterized by running, speed changes, and short-distance acceleration, the neuromuscular stimuli generated by repeated high-intensity actions may produce a small transfer effect on jump performance by increasing neuromuscular activation, enhancing rapid force production in the lower limbs, and optimizing the efficiency of stretch-shortening cycle utilization (Markström and Olsson, 2013; Tillin et al., 2013; Uthoff et al., 2020). This may also be because trained adolescent athletes generally already possess a certain foundation of strength and power, and further improvements in CMJ may require more targeted neuromuscular stimuli, such as resistance training, plyometric training, or their combination (Haghighi et al., 2023; Hammami et al., 2021; Oliver et al., 2024).

Neither the subgroup analyses nor the exploratory univariable meta-regression provided clear evidence that age, intervention duration, or training frequency influenced the effect. However, these findings should be interpreted cautiously because the analyses were based on study-level data and a limited number of comparisons.

### 4.3 Sprint performance

HIIT-based interventions improved sprint performance in trained adolescent athletes, though their training effects were not entirely consistent across sprint distances. For the ≤10m sprint, the magnitude of improvement reached a moderate level, whereas for the ≥20m sprint, the magnitude of improvement was relatively small. Overall, HIIT-based interventions may promote sprint performance by improving neuromuscular activation, rapid force production, and force output during the acceleration phase. Among these outcomes, all studies included in the ≤10m sprint analysis used running-based HIIT. Therefore, this finding mainly reflects the effect of running-based HIIT on short-distance acceleration ability.

This may also partly explain the difference in effect magnitude between sprint distances. Performance over ≤10m depends more on rapid force production during the starting phase, neuromuscular recruitment efficiency, and horizontal acceleration ability (Standing and Maulder, 2017; Yu et al., 2016). Running-based HIIT, especially when sprint-oriented, typically involves repeated short-distance accelerations and may therefore closely match the demands of short-distance sprinting by improving horizontal force output, rapid force production, and neuromuscular recruitment efficiency (Fessi et al., 2018; Thurlow et al., 2025).

In contrast, ≥20m sprint depends not only on acceleration ability but also more strongly on maintaining maximal speed, coordination of stride frequency and stride length, and high-speed running technique (Chatzilazaridis et al., 2012; von Lieres und Wilkau et al., 2020). These qualities are more likely to require the more specific stimuli provided by sprint-oriented training, which may help explain why the improvement in ≥ 20m sprint performance remained relatively small in the present review (Myrvang and van den Tillaar, 2024; Wild et al., 2011; Young et al., 2018).

The subgroup analyses in this study further showed a significant between-subgroup difference in competitive level for ≥20m sprint performance. Higher-level athletes usually have greater accumulated training exposure, more mature sprint-related mechanical characteristics, and more refined running technique (Keiner et al., 2021; Oliver et al., 2024). Accordingly, it is possible that the adaptations induced by HIIT-based interventions may translate more readily into improvements in ≥20m sprint performance. However, this exploratory finding should not be interpreted as definitive evidence of a competitive-level moderator effect.

### 4.4 Change-of-direction

HIIT-based interventions significantly improved COD in trained adolescent athletes, although the magnitude of improvement was relatively small. This may be partly related to improved rapid force production and movement control during the braking-to-reacceleration phase (Kinnunen et al., 2019; Kızılca et al., 2026). However, COD depends not only on physical factors such as acceleration, deceleration, eccentric control, and lower-limb strength, but also on movement technique, center-of-mass control, and task context (Jones et al., 2009; Singh et al., 2025). Therefore, the training stimulus provided by HIIT-based interventions alone may be insufficient to meet the specific technical and mechanical demands required for COD improvement, and its effect on COD may therefore be relatively limited. Previous studies have also suggested that adding COD sprint training to HIIT-based interventions may be more helpful for improving COD, because this type of training is closer to the actual running and direction-change demands seen in sport (Selmi et al., 2026; Stanković et al., 2024).

The overall heterogeneity of COD in this study was also low, which suggests that this outcome was fairly consistent across studies. The subgroup analyses further showed no significant between-subgroup differences for age, competitive level, intervention duration, training frequency, total number of training sessions, HIIT type, or HIIT mode.

### 4.5 Repeated sprint ability

RST significantly improved RSA in trained adolescent athletes, with a moderate magnitude of improvement. Because all training protocols included in the RSA analysis in this study were RST, the present findings should be interpreted more specifically as evidence that RST has a positive effect on RSA in trained adolescent athletes, rather than being simply generalized to all HIIT types. RSA is not a single physical fitness component, but rather a composite performance determined jointly by single-sprint ability and the capacity to recover between sprints (Bishop et al., 2011; Girard et al., 2011). Its development and expression are influenced by phosphocreatine resynthesis, anaerobic and aerobic energy supply processes, and neuromuscular function (Collins et al., 2018; Milioni et al., 2017).

RST may improve RSA through repeated short-duration sprint efforts with incomplete recovery, which can simultaneously stimulate anaerobic energy supply and aerobic recovery systems and may also improve neuromuscular function to some extent, thereby enhancing the ability to maintain output during consecutive sprints (Bishop et al., 2011; Gharbi et al., 2015; Thurlow et al., 2023). Because RST and RSA show a high degree of correspondence in movement pattern, energy metabolism characteristics, and recovery demands, the training adaptations induced by RST may have relatively strong specificity of transfer (Bishop et al., 2011; Bravo et al., 2008; Thurlow et al., 2024). This may also help explain why RSA showed a moderate magnitude of improvement in the present study. Given the current limited evidence, future research is needed to clarify the effects of different HIIT types on RSA.

### 4.6 Practical applications

From a practical perspective, HIIT-based interventions may be an efficient training option when the primary goal is to improve aerobic fitness, intermittent running performance, and short-distance acceleration in trained adolescent athletes. By contrast, HIIT-based interventions should not be viewed as a stand-alone solution for maximizing CMJ, ≥20m sprint performance, or COD, because these outcomes likely require more targeted strength, plyometric, sprint, or COD-specific training. For RSA, the current evidence more specifically supports the use of RST. Therefore, HIIT-based interventions are best incorporated as one part of an integrated physical training program, with its specific role adjusted according to sport demands, season phase, total training load, and athlete recovery status.

## 5 Limitations

This study has several limitations that should be acknowledged. Substantial differences across studies in participant characteristics, training protocols, testing methods, and comparator conditions may have reduced the comparability of the pooled estimates and limited our ability to determine the optimal training prescription. In addition, comparator conditions included regular sport-specific training as well as various non-HIIT programs, such as coordination training, continuous training, and resistance training, and were unevenly represented across outcomes. Therefore, comparator-stratified analyses were not performed, and we were unable to determine whether the pooled effects differed according to the type of comparison condition. The number of included studies was relatively small for some outcomes and subgroup analyses, which reduced statistical power and means that these findings should be interpreted cautiously. The RSA findings were derived mainly from RST protocols and therefore should not be generalized to all HIIT types. Only peer-reviewed studies published in English were included, which may have led to the omission of relevant evidence. In some studies, change-score standard deviations had to be estimated rather than obtained directly, which may have introduced additional uncertainty. Methodological limitations in several included studies, particularly in randomization, allocation concealment, and blinding, may have increased the risk of bias, and the certainty of evidence for some outcomes remained low or very low. Finally, the included studies were dominated by soccer samples, which may limit the generalizability of the findings to other sports. Accordingly, the findings of this review should be interpreted with caution, and more high-quality randomized controlled trials across a broader range of sports are still needed.

## 6 Conclusion

This systematic review and meta-analysis suggest that, compared with non-HIIT comparator conditions, HIIT-based interventions may improve several physical fitness outcomes in trained adolescent athletes. Among these outcomes, the improvement in VO_2_max was moderate. For field-based intermittent endurance test performance, VIFT, and ≤10m sprint, the pooled analyses also showed moderate improvements. By contrast, the magnitude of improvement in CMJ, ≥20m sprint, and change-of-direction ability was relatively small. For RSA, the observed improvement was mainly driven by RST, with a moderate magnitude. Overall, HIIT-based interventions may be a useful training option for improving selected physical fitness outcomes in trained adolescent athletes.

## Data Availability

The original contributions presented in the study are included in the article/**Supplementary Material**. Further inquiries can be directed to the corresponding author.
