# Supplementary material for: Effects of high-intensity interval training on physical fitness in trained adolescent athletes: a systematic review and meta-analysis
Source: Front Physiol. 2026 May 18;17:1839190. doi: 10.3389/fphys.2026.1839190 (PMC13222820; doi:10.3389/fphys.2026.1839190)
Supplement: Supplementary file 1 [file DataSheet1.docx]

Table S1. Full electronic search strategies for all databases.

| **Database** | **Complete Search Strategy** | **Hits** |
| --- | --- | --- |
| Web of Science | TS=("high intensity interval training" OR HIIT OR "high-intensity interval" OR "high intensity interval" OR "interval training" OR "sprint interval training" OR SIT OR "repeated sprint training" OR "repeated-sprint training" OR RST) AND TS=(adolescen* OR teen* OR youth* OR "young athlete*" OR "youth athlete*") AND TS=(athlete* OR sport* OR soccer OR football OR basketball OR handball OR volleyball OR rugby OR swimmer* OR runne* OR cyclist* OR tennis OR badminton OR taekwondo OR "team sport*" OR "individual sport*") | 895 |
| Scopus | TITLE-ABS-KEY("high intensity interval training" OR HIIT OR "high-intensity interval" OR "high intensity interval" OR "interval training" OR "sprint interval training" OR SIT OR "repeated sprint training" OR "repeated-sprint training" OR RST) AND TITLE-ABS-KEY(adolescen* OR teen* OR youth* OR "young athlete*" OR "youth athlete*") AND TITLE-ABS-KEY(athlete* OR sport* OR soccer OR football OR basketball OR handball OR volleyball OR rugby OR swimmer* OR runne* OR cyclist* OR tennis OR badminton OR taekwondo OR "team sport*" OR "individual sport*") | 1039 |
| PubMed | ("High-Intensity Interval Training"[Mesh] OR "high intensity interval training"[tiab] OR HIIT[tiab] OR "high-intensity interval"[tiab] OR "high intensity interval"[tiab] OR "interval training"[tiab] OR "sprint interval training"[tiab] OR SIT[tiab] OR "repeated sprint training"[tiab] OR "repeated-sprint training"[tiab] OR RST[tiab]) AND ("Adolescent"[Mesh] OR adolescen*[tiab] OR teen*[tiab] OR youth*[tiab] OR "young athlete*"[tiab] OR "youth athlete*"[tiab]) AND ("Athletes"[Mesh] OR athlete*[tiab] OR sport*[tiab] OR soccer[tiab] OR football[tiab] OR basketball[tiab] OR handball[tiab] OR volleyball[tiab] OR rugby[tiab] OR swimmer*[tiab] OR runne*[tiab] OR cyclist*[tiab] OR tennis[tiab] OR badminton[tiab] OR taekwondo[tiab] OR "team sport*"[tiab] OR "individual sport*"[tiab]) | 618 |
| Embase | ('high intensity interval training'/exp OR 'high intensity interval training':ti,ab,kw OR hiit:ti,ab,kw OR 'high-intensity interval':ti,ab,kw OR 'high intensity interval':ti,ab,kw OR 'interval training':ti,ab,kw OR 'sprint interval training':ti,ab,kw OR sit:ti,ab,kw OR 'repeated sprint training':ti,ab,kw OR 'repeated-sprint training':ti,ab,kw OR rst:ti,ab,kw) AND ('adolescent'/exp OR adolescen*:ti,ab,kw OR teen*:ti,ab,kw OR youth*:ti,ab,kw OR 'young athlete*':ti,ab,kw OR 'youth athlete*':ti,ab,kw) AND ('athlete'/exp OR athlete*:ti,ab,kw OR sport*:ti,ab,kw OR soccer:ti,ab,kw OR football:ti,ab,kw OR basketball:ti,ab,kw OR handball:ti,ab,kw OR volleyball:ti,ab,kw OR rugby:ti,ab,kw OR swimmer*:ti,ab,kw OR runne*:ti,ab,kw OR cyclist*:ti,ab,kw OR tennis:ti,ab,kw OR badminton:ti,ab,kw OR taekwondo:ti,ab,kw OR 'team sport*':ti,ab,kw OR 'individual sport*':ti,ab,kw) | 718 |
| Cochrane Library | (("high intensity interval training" OR HIIT OR "high-intensity interval" OR "high intensity interval" OR "interval training" OR "sprint interval training" OR SIT OR "repeated sprint training" OR "repeated-sprint training" OR RST)):ti,ab,kw AND ((adolescen* OR teen* OR youth* OR "young athlete*" OR "youth athlete*")):ti,ab,kw AND ((athlete* OR sport* OR soccer OR football OR basketball OR handball OR volleyball OR rugby OR swimmer* OR runne* OR cyclist* OR tennis OR badminton OR taekwondo OR "team sport*" OR "individual sport*")):ti,ab,kw | 365 |

Table S2. Subgroup analysis results for VO₂max.

| **Subgroup** | **Level** | **K** | **n** | **ES (95% CI)** | **p (Overall Effect)** | **I² (%)** | **p (Heterogeneity)** | **p (Subgroup Difference)** |
| --- | --- | --- | --- | --- | --- | --- | --- | --- |
| **Maximal oxygen uptake (VO₂max)** | | | | | | | | |
| Age | >16.38 | 7 | 221 | 0.71 (0.41, 1.00) | <0.001 | 0.0 | 0.887 | 0.642 |
|  | ≤16.38 | 10 | 301 | 0.61 (0.35, 0.87) | <0.001 | 13.1 | 0.316 |  |
| Competitive level | Tier 3 | 8 | 232 | 0.77 (0.49, 1.05) | <0.001 | 0.0 | 0.701 | 0.255 |
|  | Tier 2 | 8 | 290 | 0.55 (0.30, 0.80) | <0.001 | 0.0 | 0.603 |  |
| Training duration | ≥8 | 9 | 267 | 0.49 (0.24, 0.75) | <0.001 | 0.0 | 0.456 | 0.084 |
|  | <8 | 7 | 255 | 0.82 (0.55, 1.10) | <0.001 | 0.0 | 0.938 |  |
| Training frequency | ≤2.0 | 11 | 351 | 0.67 (0.43, 0.91) | <0.001 | 6.8 | 0.375 | 0.722 |
|  | >2.0 | 5 | 171 | 0.60 (0.27, 0.92) | <0.001 | 0.0 | 0.930 |  |
| Total sessions | >13.0 | 8 | 256 | 0.53 (0.24, 0.83) | <0.001 | 18.4 | 0.269 | 0.277 |
|  | ≤13.0 | 8 | 266 | 0.75 (0.49, 1.02) | <0.001 | 0.0 | 0.949 |  |
| HIIT type | Interval | 8 | 185 | 0.68 (0.36, 1.00) | <0.001 | 0.0 | 0.852 | 0.876 |
|  | RST | 5 | 169 | 0.55 (0.10, 1.00) | 0.017 | 45.0 | 0.091 |  |
|  | SIT | 4 | 168 | 0.68 (0.35, 1.01) | <0.001 | 0.0 | 0.848 |  |
| HIIT mode | Running-based HIIT | 12 | 359 | 0.68 (0.44, 0.92) | <0.001 | 12.5 | 0.316 | 0.655 |
|  | Sport-specific HIIT | 6 | 163 | 0.58 (0.24, 0.93) | <0.001 | 0.0 | 0.933 |  |

Note: Because multi-arm trials may contribute to different HIIT categories, the summed K across subgroups may exceed the overall K.

Table S3. Subgroup analysis results for Field-based intermittent endurance test performance.

| **Subgroup** | **Level** | **K** | **n** | **ES (95% CI)** | **p (Overall Effect)** | **I² (%)** | **p (Heterogeneity)** | **p (Subgroup Difference)** |
| --- | --- | --- | --- | --- | --- | --- | --- | --- |
| Field-based intermittent endurance test performance | | | | | | | | |
| Age | <16.00 | 3 | 85 | -0.09 (-0.95, 0.76) | 0.837 | 69.3 | 0.020 | 0.033 |
|  | ≥16.00 | 6 | 203 | 1.07 (0.43, 1.70) | 0.001 | 74.4 | <0.001 |  |
| Competitive level | Tier 2 | 7 | 234 | 0.37 (-0.15, 0.88) | 0.159 | 69.2 | 0.001 | 0.104 |
|  | Tier 3 | 2 | 54 | 2.18 (0.05, 4.31) | 0.045 | 88.5 | 0.003 |  |
| Training duration | ≥8.0 | 5 | 175 | 0.20 (-0.50, 0.91) | 0.578 | 77.9 | <0.001 | 0.076 |
|  | <8.0 | 4 | 113 | 1.25 (0.33, 2.18) | 0.008 | 77.3 | 0.001 |  |
| Total sessions | ≥16.0 | 5 | 175 | 0.20 (-0.50, 0.91) | 0.578 | 77.9 | <0.001 | 0.076 |
|  | <16.0 | 4 | 113 | 1.25 (0.33, 2.18) | 0.008 | 77.3 | 0.001 |  |
| HIIT type | SIT | 2 | 76 | 0.45 (-1.11, 2.01) | 0.572 | 90.5 | 0.001 | 0.224 |
|  | RST | 3 | 88 | 0.18 (-0.56, 0.93) | 0.636 | 61.2 | 0.036 |  |
|  | Interval | 4 | 124 | 1.34 (0.26, 2.42) | 0.015 | 84.7 | <0.001 |  |
| HIIT mode | Running-based HIIT | 7 | 243 | 0.66 (-0.03, 1.36) | 0.063 | 82.5 | <0.001 | 0.916 |
|  | Sport-specific HIIT | 2 | 45 | 0.60 (-0.43, 1.63) | 0.254 | 64.7 | 0.092 |  |

Note: Because multi-arm trials may contribute to different HIIT categories, the summed K across subgroups may exceed the overall K.

Table S4. Subgroup analysis results for CMJ.

| **Subgroup** | **Level** | **K** | **n** | **ES (95% CI)** | **p (Overall Effect)** | **I² (%)** | **p (Heterogeneity)** | **p (Subgroup Difference)** |
| --- | --- | --- | --- | --- | --- | --- | --- | --- |
| **Countermovement jump (CMJ)** | | | | | | | | |
| Age | >16.15 | 9 | 303 | 0.73 (0.19, 1.28) | 0.009 | 77.3 | <0.001 | 0.101 |
|  | ≤16.15 | 11 | 240 | 0.23 (-0.03, 0.49) | 0.083 | 0.0 | 0.490 |  |
| Competitive level | Tier 3 | 12 | 301 | 0.48 (-0.03, 0.99) | 0.065 | 76.0 | <0.001 | 0.750 |
|  | Tier 2 | 8 | 242 | 0.38 (0.12, 0.65) | 0.005 | 0.0 | 0.586 |  |
| Training duration | ≥8 | 12 | 345 | 0.38 (0.09, 0.67) | 0.010 | 41.3 | 0.059 | 0.607 |
|  | <8 | 8 | 198 | 0.57 (-0.08, 1.22) | 0.086 | 75.8 | <0.001 |  |
| Training frequency | ≤2.0 | 14 | 385 | 0.45 (0.04, 0.86) | 0.031 | 72.1 | <0.001 | 0.985 |
|  | >2.0 | 6 | 158 | 0.44 (0.08, 0.81) | 0.018 | 10.9 | 0.346 |  |
| Total sessions | >13.5 | 11 | 343 | 0.69 (0.20, 1.19) | 0.006 | 77.5 | <0.001 | 0.056 |
|  | ≤13.5 | 9 | 200 | 0.14 (-0.16, 0.43) | 0.352 | 0.0 | 0.681 |  |
| HIIT type | Interval | 10 | 237 | 0.41 (-0.19, 1.00) | 0.177 | 78.0 | <0.001 | 0.595 |
|  | RST | 7 | 175 | 0.36 (-0.14, 0.85) | 0.154 | 58.0 | 0.020 |  |
|  | SIT | 4 | 131 | 0.65 (0.28, 1.02) | <0.001 | 0.0 | 0.934 |  |
| HIIT mode | Running-based HIIT | 15 | 391 | 0.43 (0.01, 0.84) | 0.042 | 73.0 | <0.001 | 0.823 |
|  | Sport-specific HIIT | 6 | 152 | 0.49 (0.14, 0.83) | 0.005 | 0.0 | 0.567 |  |

Note: Because multi-arm trials may contribute to different HIIT categories, the summed K across subgroups may exceed the overall K.

Table S5. Subgroup analysis results for ≥20 m sprint.

| **Subgroup** | **Level** | **K** | **n** | **ES (95% CI)** | **p (Overall Effect)** | **I² (%)** | **p (Heterogeneity)** | **p (Subgroup Difference)** |
| --- | --- | --- | --- | --- | --- | --- | --- | --- |
| **≥20 m sprint** | | | | | | | | |
| Age | ≤16.24 | 10 | 205 | -0.24 (-0.52, 0.04) | 0.093 | 0.0 | 0.609 | 0.711 |
|  | >16.24 | 8 | 218 | -0.31 (-0.58, -0.04) | 0.024 | 0.0 | 0.449 |  |
| Competitive level | Tier 3 | 10 | 242 | -0.47 (-0.75, -0.20) | <0.001 | 7.8 | 0.370 | 0.034 |
|  | Tier 2 | 8 | 181 | -0.04 (-0.33, 0.24) | 0.783 | 0.0 | 1.000 |  |
| Sprint distance | 20 m | 11 | 268 | -0.36 (-0.60, -0.12) | 0.003 | 0.0 | 0.550 | 0.262 |
|  | >20 m | 7 | 155 | -0.13 (-0.45, 0.19) | 0.426 | 0.0 | 0.640 |  |
| Training duration | >8 | 6 | 164 | -0.20 (-0.52, 0.11) | 0.213 | 0.0 | 0.789 | 0.567 |
|  | ≤8 | 12 | 259 | -0.32 (-0.57, -0.07) | 0.012 | 6.2 | 0.384 |  |
| Training frequency | ≤2 | 11 | 262 | -0.35 (-0.59, -0.10) | 0.005 | 0.1 | 0.445 | 0.356 |
|  | >2 | 7 | 161 | -0.16 (-0.47, 0.15) | 0.312 | 0.0 | 0.754 |  |
| Total sessions | >17 | 9 | 241 | -0.24 (-0.50, 0.02) | 0.070 | 0.0 | 0.777 | 0.759 |
|  | ≤17 | 9 | 182 | -0.31 (-0.62, 0.01) | 0.054 | 14.5 | 0.309 |  |
| HIIT type | RST | 9 | 228 | -0.42 (-0.71, -0.14) | 0.004 | 14.9 | 0.302 | 0.214 |
|  | Interval | 7 | 131 | -0.18 (-0.52, 0.17) | 0.306 | 0.0 | 0.982 |  |
|  | SIT | 2 | 64 | 0.06 (-0.43, 0.55) | 0.810 | 0.0 | 0.896 |  |
| HIIT mode | Running-based HIIT | 14 | 334 | -0.31 (-0.53, -0.09) | 0.006 | 0.0 | 0.475 | 0.517 |
|  | Sport-specific HIIT | 4 | 89 | -0.15 (-0.57, 0.26) | 0.479 | 0.0 | 0.754 |  |

Note: Because multi-arm trials may contribute to different HIIT categories, the summed K across subgroups may exceed the overall K.

Table S6. Subgroup analysis results for COD.

| **Subgroup** | **Level** | **K** | **n** | **ES (95% CI)** | **p (Overall Effect)** | **I² (%)** | **p (Heterogeneity)** | **p (Subgroup Difference)** |
| --- | --- | --- | --- | --- | --- | --- | --- | --- |
| **Change-of-direction (COD)** | | | | | | | | |
| Age | <16.1 | 9 | 211 | -0.56 (-0.84 to -0.28) | <0.001 | 0.0 | 0.860 | 0.923 |
|  | ≥16.1 | 11 | 339 | -0.54 (-0.81 to -0.27) | <0.001 | 24.5 | 0.177 |  |
| Competitive level | Tier 3 | 11 | 289 | -0.63 (-0.93 to -0.33) | <0.001 | 31.2 | 0.127 | 0.367 |
|  | Tier 2 | 9 | 261 | -0.45 (-0.71 to -0.19) | <0.001 | 0.0 | 0.944 |  |
| Training duration | <8 | 7 | 175 | -0.68 (-1.01 to -0.35) | <0.001 | 0.0 | 0.711 | 0.344 |
|  | ≥8 | 13 | 375 | -0.49 (-0.72 to -0.26) | <0.001 | 13.6 | 0.298 |  |
| Training frequency | ≤2 | 10 | 289 | -0.52 (-0.81 to -0.23) | <0.001 | 26.2 | 0.180 | 0.814 |
|  | >2 | 10 | 261 | -0.57 (-0.83 to -0.31) | <0.001 | 0.0 | 0.764 |  |
| Total sessions | ≤16 | 10 | 265 | -0.61 (-0.90 to -0.33) | <0.001 | 12.7 | 0.314 | 0.499 |
|  | >16 | 10 | 285 | -0.48 (-0.73 to -0.24) | <0.001 | 0.0 | 0.604 |  |
| HIIT type | RST | 5 | 161 | -0.77 (-1.26 to -0.29) | 0.002 | 54.5 | 0.031 | 0.100 |
|  | Interval | 12 | 258 | -0.33 (-0.58 to -0.08) | 0.010 | 0.0 | 0.997 |  |
|  | SIT | 4 | 131 | -0.76 (-1.17 to -0.35) | <0.001 | 0.0 | 0.968 |  |
| HIIT mode | Running-based HIIT | 12 | 332 | -0.57 (-0.85 to -0.28) | <0.001 | 34.4 | 0.100 | 0.834 |
|  | Sport-specific HIIT | 9 | 218 | -0.52 (-0.81 to -0.24) | <0.001 | 0.0 | 0.943 |  |

Note: Because multi-arm trials may contribute to different HIIT categories, the summed K across subgroups may exceed the overall K.

Table S7. Subgroup analysis results for RSA.

| **Subgroup** | **Level** | **K** | **n** | **ES (95% CI)** | **p (Overall Effect)** | **I² (%)** | **p (Heterogeneity)** | **p (Subgroup Difference)** |
| --- | --- | --- | --- | --- | --- | --- | --- | --- |
| Repeated sprint ability (RSA) | | | | | | | | |
| Age | <16.38 | 3 | 66 | -0.43 (-0.94, 0.08) | 0.098 | 0.0 | 0.714 | 0.230 |
|  | ≥16.38 | 4 | 142 | -0.85 (-1.29, -0.40) | <0.001 | 34.3 | 0.193 |  |
| Competitive level | Tier 2 | 2 | 44 | -0.35 (-0.98, 0.29) | 0.280 | 0.0 | 0.555 | 0.233 |
|  | Tier 3 | 5 | 164 | -0.81 (-1.18, -0.43) | <0.001 | 21.8 | 0.270 |  |
| Training duration | ≥8.0 | 5 | 156 | -0.60 (-0.94, -0.27) | <0.001 | 0.0 | 0.481 | 0.362 |
|  | <8.0 | 2 | 52 | -1.03 (-1.89, -0.18) | 0.018 | 51.7 | 0.150 |  |
| Training frequency | <2 | 2 | 35 | -0.51 (-1.19, 0.17) | 0.142 | 0.0 | 0.521 | 0.574 |
|  | ≥2 | 5 | 173 | -0.74 (-1.13, -0.35) | <0.001 | 28.2 | 0.213 |  |
| Total sessions | ≥16 | 3 | 121 | -0.62 (-1.06, -0.18) | 0.006 | 20.1 | 0.287 | 0.575 |
|  | <16 | 4 | 87 | -0.81 (-1.31, -0.30) | 0.002 | 21.1 | 0.283 |  |

Note: Because multi-arm trials may contribute to different HIIT categories, the summed K across subgroups may exceed the overall K.

Table S8. Exploratory univariable meta-regression analyses for CMJ.

| **Covariate** | **Coefficient** | **Lower 95% CI** | **Upper 95% CI** | **p** |
| --- | --- | --- | --- | --- |
| Age | 0.214 | -0.044 | 0.472 | 0.099 |
| Intervention duration | -0.005 | -0.138 | 0.128 | 0.936 |
| Training frequency | 0.019 | -0.610 | 0.648 | 0.950 |

Figure S1. Leave-one-out sensitivity analysis for VO₂max.

Figure S2. Leave-one-out sensitivity analysis for field-based intermittent endurance test performance.

Figure S3. Leave-one-out sensitivity analysis for VIFT.

Figure S4. Leave-one-out sensitivity analysis for CMJ.

Figure S5. Leave-one-out sensitivity analysis for ≤ 10 m sprint.

Figure S6. Leave-one-out sensitivity analysis for ≥ 20 m sprint.

Figure S7. Leave-one-out sensitivity analysis for COD.

Figure S8. Leave-one-out sensitivity analysis for RSA.
